# Photophysical Studies of a Zr(IV) Complex with Two Pyrrolide-Based Tetradentate Schiff Base Ligands

**DOI:** 10.1021/acs.inorgchem.4c00365

**Published:** 2024-05-03

**Authors:** Yu Zhang, Tia S. Lee, Jeffrey L. Petersen, Carsten Milsmann

**Affiliations:** † C. Eugene Bennett Department of Chemistry, West Virginia University, Morgantown, West Virginia 26506, United States; ‡ Department of Chemistry, Tufts University, Medford, Massachusetts 02144, United States; § Department of Chemistry, Princeton University, Princeton, New Jersey 08544, United States

## Abstract

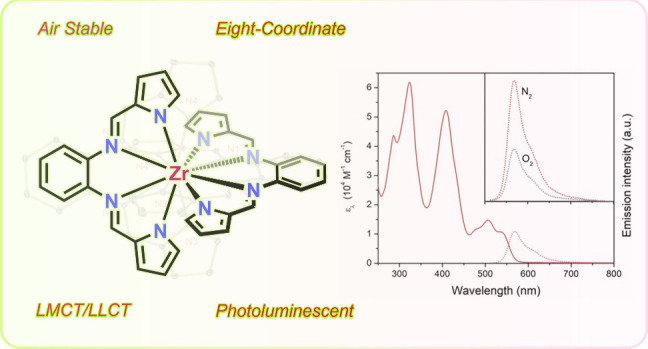

The reaction of two equivalents of *N*,*N’*-bis(2-pyrrolylmethylidene)-1,2-phenylenediamine (H_2_bppda) with tetrabenzylzirconium provided the air- and moisture-stable eight-coordinate complex Zr(bppda)_2_. Temperature-dependent steady-state and time-resolved emission spectroscopy established weak photoluminescence (Φ_PL_ = 0.4% at 293 K) by a combination of prompt fluorescence and thermally activated delayed fluorescence (TADF) upon visible light excitation at and around room temperature. TADF emission is strongly quenched by ^3^O_2_ and shows highly temperature-sensitive emission lifetimes of hundreds of microseconds. The lifetime of the lowest energy singlet excited state, S_1_, was established by transient absorption spectroscopy and shows rapid deactivation (τ = 142 ps) by prompt fluorescence and intersystem crossing to the triplet state, T_1_. Time-dependent density functional theory (TD-DFT) calculations predict moderate ligand-to-metal charge transfer (LMCT) contributions of 25–30% for the S_1_ and T_1_ states. A comparison of Zr(bppda)_2_ to related zirconium pyridine dipyrrolide complexes, Zr(PDP)_2_, revealed important electronic structure changes due to the eight-coordinate ligand environment in Zr(bppda)_2_, which were correlated to differences in the photophysical properties between the two compound classes.

## Abstract 2

The eight-coordinate zirconium complex Zr(bppda)_2_ exhibits photoluminescence through a combination of prompt fluorescence and thermally activated delayed fluorescence (TADF) at room temperature. The lowest energy singlet and triplet excited states contain significant ligand-to-metal charge transfer character involving the d^0^ metal ion. Femtosecond transient absorption spectroscopy revealed slower intersystem crossing rates compared to related Zr(PDP)_2_ complexes, which were correlated with subtle changes in the electronic structures of the two compound classes.

## Introduction

Photoactive transition metal complexes based on Earth-abundant elements have become the focus of intense research over the past decade.^1−7^ Among them, photoluminescent early transition metal complexes displaying long-lived excited states are an emerging class of inorganic chromophores that have found application in photoredox catalysis,^8−11^ photon upconversion,^12^ and biological imaging and sensing.^13,14^ Due to the relatively high abundance of early transition metals in the Earth’s crust,^15^ these photoactive complexes are an attractive alternative to precious metal chromophores, which have traditionally dominated the field of molecular inorganic photochemistry.^16−19^ While this offers the prospect of more sustainable and cost-effective photochemical applications, it also provides unique opportunities to challenge existing paradigms in photochemistry and expand the fundamental understanding of different excited state manifolds.^1−7^ When combined with electron-rich ligand scaffolds, the electron-poor nature of early transition metals provides ideal conditions for the generation of low energy excited states with significant ligand-to-metal charge transfer (LMCT) character, which have historically been underexplored in transition metal photochemistry and photophysics.^20−22^ The strong preference for d^0^ electron configurations in early transition metals eliminates potentially detrimental metal-centered (MC) excited states, often resulting in remarkably long photoluminescence lifetimes of tens to hundreds of microseconds at room temperature in solution. While initial examples for d^0^ LMCT luminophores relied heavily on group 3 and 4 metallocenes^23−34^ or group 5 imido complexes,^35,36^ more recent reports introduced complexes with multiple pincer-type pyridine dipyrrolide^8−11,14,37^ and bis(aryloxide) N-heterocyclic carbene ligands^38^ or bidentate 2-(2′-pyridine)pyrrolide ligands.^39,40^ These ligand architectures provide increased synthetic modularity and, thereby, promise better control over the photophysical properties.

**Figure 1:**
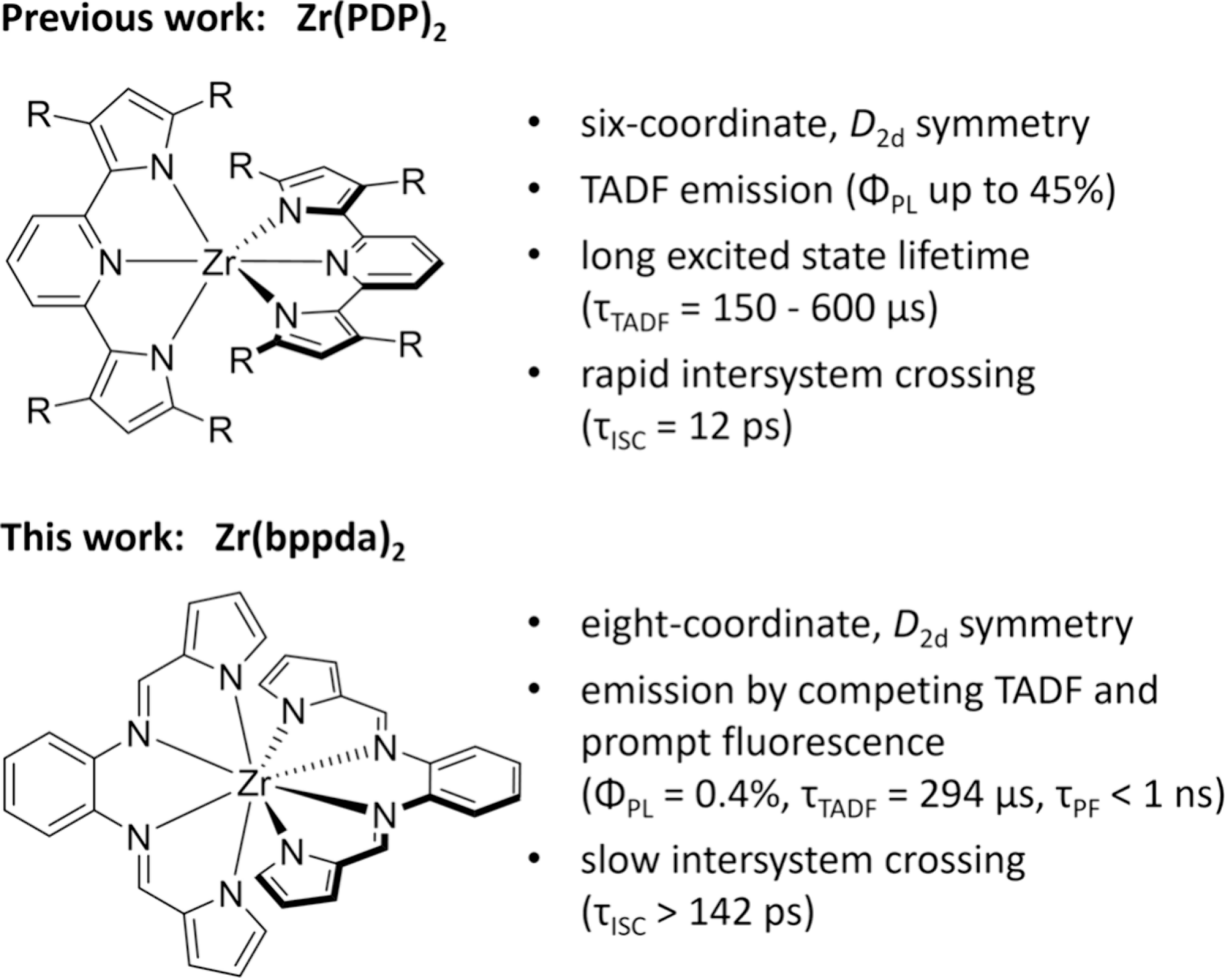
Comparison of photoluminescent Zr(PDP)_2_ and Zr(bppda)_2_ complexes.

We previously reported that bis(pyridine dipyrrolide)zirconium complexes, Zr(PDP)_2_, exhibit efficient and remarkably long-lived photoluminescence by thermally activated delayed fluorescence (TADF) emanating from excited states with significant LMCT character (Figure 1).^11^ Inspired by these results, we were curious whether other planar ligand architectures with multiple electron-rich pyrrolide heterocycles incorporated into a fully conjugated π-system could be utilized to generate photoluminescent zirconium complexes. A promising candidate was identified in *N*,*N’*-bis(2-pyrrolylmethylidene)-1,2-phenylenediamine (H_2_bppda), which replaces the central pyridine ring of pyridine dipyrrolide ligands with a phenylenediamine unit.^41,42^ As an analog to well-studied *N*,*N’*-bis(salicylidene)ethylenediamine (salen) and porphyrin ligands, this pyrrole-based Schiff-base ligand has found use in coordination chemistry and catalysis but has not been explored for the generation of photoluminescent metal complexes.^42−52^ Straightforward synthetic access through a simple condensation of commercially available pyrrole-2-carboxaldehyde and 1,2-phenylenediamine was seen as an additional benefit of the H_2_bppda framework.^53^

Here we report the synthesis and characterization of the eight-coordinate, photoluminescent zirconium complex Zr(bppda)_2_, where [bppda]^2–^ is the doubly deprotonated form of *N,N’*-bis(2-pyrrolylmethylidene)-1,2-phenylenediamine. Photophysical studies of air- and moisture stable Zr(bppda)_2_ establish emission by a combination of prompt and delayed fluorescence at and around room temperature in solution. Computational studies by time-dependent density functional theory (TD-DFT) provide insight into the nature of the excited states and reveal moderate LMCT contributions. The comparison of Zr(bppda)_2_ to Zr(PDP)_2_ complexes establishes subtle differences in their electronic structures, which can be related to changes in the optical properties, providing deeper insight into both compound classes.

## Results and Discussion

### Synthesis and Characterization of Zr(bppda)_2_

**Scheme 1:**
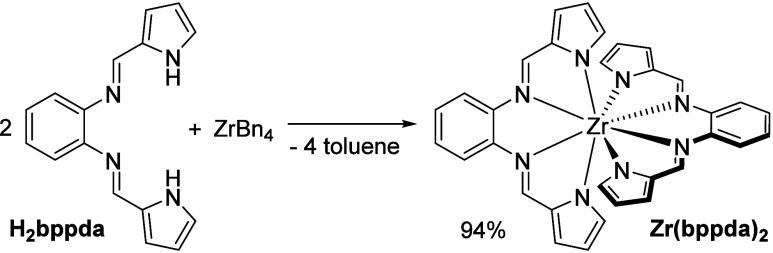
Synthesis of Zr(bppda)_2_

The straightforward room-temperature reaction of two equivalents of H_2_bppda with tetrabenzylzirconium, ZrBn_4_, or tetrakis(dimethylamido)zirconium, Zr(NMe_2_)_4_, in benzene provided analytically pure Zr(bppda)_2_ as a microcrystalline, red-orange precipitate in near-quantitative yields within 30 min (Scheme 1).

**Figure 2:**
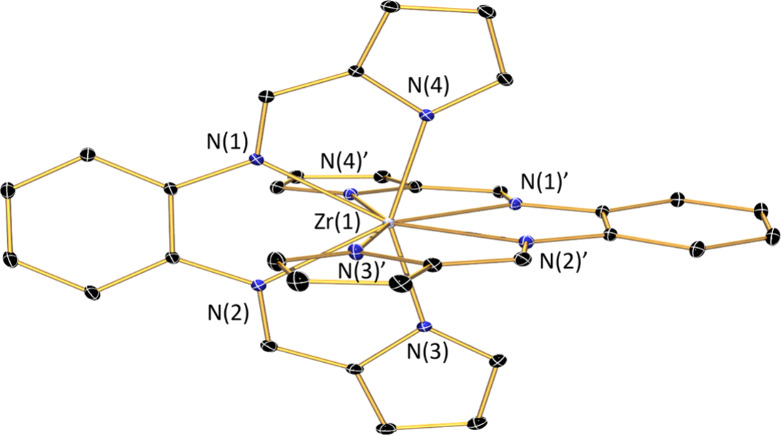
Representation of the solid-state molecular structure of Zr(bppda)_2_ at 30% probability ellipsoids. Hydrogen atoms and a cocrystallized molecule of benzene are omitted for clarity.

The molecular structure of Zr(bppda)_2_ was established by X-ray crystallography (Figure 2), and important bond lengths and angles are summarized in Table 1. Like in the structure of the previously reported titanium analog Ti(bppda)_2_,^49^ two meridionally coordinating tetradentate [bppda]^2–^ ligands create an eight-coordinate environment around the central metal ion of Zr(bppda)_2_. The molecule lies on a crystallographic 2-fold rotation axis that contains the central Zr atom and passes between the pyrrolide rings of the two [bppda]^2–^ units, rendering the two ligands identical. The solid-state structure of Zr(bppda)_2_ shows only minor deviations from idealized *D*_2*d*_ symmetry, with a nearly orthogonal arrangement of the two ligands. This is reflected in a dihedral angle of 89.20(6)° between the two planes defined by the four nitrogen atoms of each ligand. Each ligand is almost perfectly planar, resulting in a sum of the four N–Zr–N angles of 360.03(4)°. The largest deviation from planarity is observed for the two carbons in the 3- and 6-positions of the phenylene backbone which are displaced by 0.159(2) Å and 0.150(2) Å above and below the plane, respectively, while the deviation for all remaining atoms is <0.1 Å. The NMR spectroscopic data for diamagnetic Zr(bppda)_2_ recorded in benzene-*d*_6_ are consistent with a similar *D*_2*d*_-symmetric structure on the NMR time scale in solution at room temperature (Figure S1).

**Table 1:** Selected Bond Lengths (Å) and Angles (deg) for Zr(bppda)_2_ Obtained by X-ray Crystallography and DFT Geometry Optimization

Zr(bppda)_2_	X-ray	DFT^a^
Zr(1)–N(1)	2.3645(12)	2.395
Zr(1)–N(2)	2.3429(11)	2.395
Zr(1)–N(3)	2.2652(13)	2.300
Zr(1)–N(4)	2.2658(13)	2.300
N(1)–Zr(1)–N(2)	66.88(4)	66.42
N(2)–Zr(1)–N(3)	70.52(4)	70.12
N(3)–Zr(1)–N(4)	152.37(4)	153.34
N(4)–Zr(1)–N(1)	70.26(4)	70.12
Dihedral angle	89.20	90

a *D*_2*d*_ symmetry enforced during calculations.

Beyond its facile synthesis and well-defined molecular structure, an additional attractive feature of Zr(bppda)_2_ is its resistance to hydrolysis resulting in excellent stability under regular benchtop conditions. In our hands, solid samples of Zr(bppda)_2_ could be manipulated and stored indefinitely in air without special precautions. Similarly, solutions for ^1^H NMR spectroscopy prepared in air using nonanhydrous benzene-*d*_6_ and 1,3,5-trimethoxybenzene as an internal standard showed no sign of decomposition after 60 days. Even the addition of water to optically dilute solutions of Zr(bppda)_2_ in THF produced no significant changes after 2 h as established by UV/vis spectroscopy (Figure S4). Minor changes were observed under these conditions after extended periods of time, but more than 90% of the original absorption intensity was retained even after 16 h. This robustness toward hydrolysis is in stark contrast to other Zr complexes with basic pyrrolide or amide ligands that typically undergo rapid protonolysis upon addition of water to form the corresponding pyrroles or amines and ZrO_2_. Relevant examples are photoluminescent bis(pyridinedipyrrolide)zirconium complexes, Zr(PDP)_2_, that undergo rapid decomposition under ambient conditions in solution and the solid state unless protected by large hydrophobic substituents on the pyrrolide rings. We propose that the stability of Zr(bppda)_2_ can be attributed primarily to the coordinatively saturated, eight-coordinate environment around the zirconium center that prevents nucleophilic attack on the metal by water.

### Room-Temperature Electronic Absorption and Emission Spectroscopy

**Figure 3:**
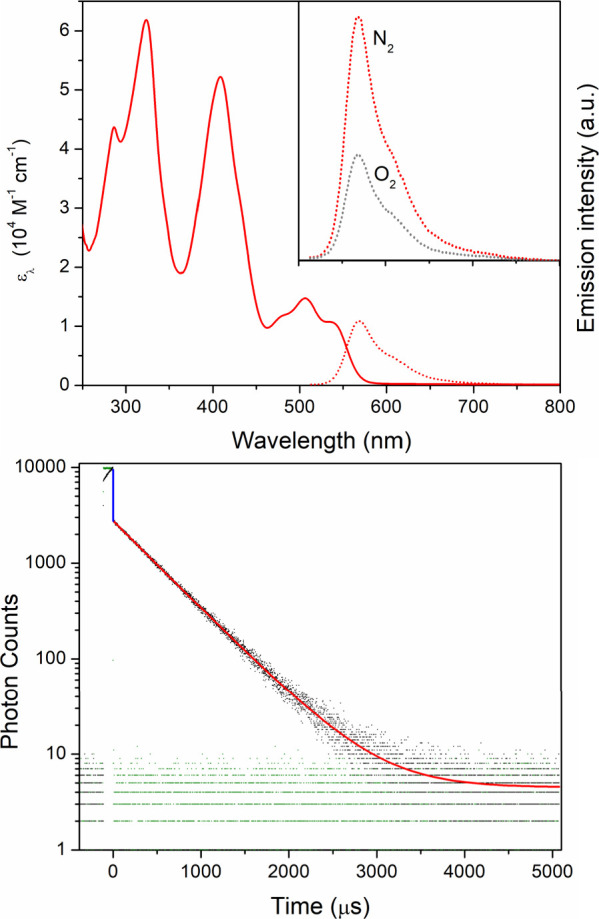
Top: Electronic absorption (solid red) and emission spectra (dotted red) of Zr(bppda)_2_ in THF under a nitrogen atmosphere. The inset shows the changes to the emission profile in the presence of molecular oxygen after exposure of the sample to air (dotted gray). Bottom: Photoluminescence decay in THF at room temperature (λ_ex_ = 516 nm). The black scatter plot represents data collected upon detection at 570 nm, and the instrument response function is shown in green. The solid blue line highlights an immediate drop in emission intensity due to rapid prompt fluorescence (τ_PL_*<* 1 ns). The solid red line represents a single-exponential fit of the long-lived emission process (τ_PL_ = 294 ± 10 μs).

The electronic absorption spectrum of Zr(bppda)_2_ recorded in THF solution at room temperature (Figure 3, top) exhibits two strong absorption bands in the UV region with maxima at 286 nm (ε_λ_ = 43,700 M^–1^ cm^–1^) and 324 nm (ε_λ_ = 61,800 M^–1^ cm^–1^). These two bands are most likely dominated by π–π* transitions within the extended π-system of the ligands as similar features can also be observed in the electronic absorption spectrum of the ligand precursor H_2_bppda (Figure S6). More importantly, two absorption features with maxima at 409 nm (ε_λ_ = 52,200 M^–1^ cm^–1^) and 506 nm (ε_λ_ = 14,700 M^–1^ cm^–1^) can be found in the visible part of the spectrum and are consistent with the intense red-orange color of the complex. Closer inspection of the band around 409 nm revealed a small shoulder at approximately 431 nm indicative of an additional unresolved feature. The lowest energy absorption maximum at 506 nm is flanked by two more clearly identifiable shoulders at around 480 and 535 nm. This structured appearance was tentatively assigned to a vibronic progression and is consistent with coupling to a vibrational mode with ν = 1070 cm^–1^. A more detailed analysis of the nature of the optical transitions in the visible part of the spectrum is provided in the computational section (*vide infra*).

Irradiation of THF solutions of Zr(bppda)_2_ at wavelength below 550 nm resulted in photoluminescence with an emission maximum at 570 nm (Figure 3, top). The emission profile is broad but exhibits a clear shoulder around 607 nm, indicative of poorly resolved vibrational fine structure similar to that observed for the lowest energy absorption band. A photoluminescence quantum yield, Φ_PL_, of 0.4% was determined in rigorously deaerated THF solution by the comparative method using Rhodamine 6G in ethanol as the reference. The low Φ_PL_ value indicates that excited state deactivation proceeds predominantly via nonradiative pathways. Exposure of the sample to air further reduced the emission intensity to 44% of the initial intensity under anaerobic conditions, establishing partial photoluminescence quenching by ^3^O_2_ and suggesting the possible involvement of a long-lived triplet excited state. Despite the clear changes in emission intensity, the peak maxima and line shapes of the emission profiles under inert atmosphere and in the presence of air are identical, and normalization of the two data sets provides superimposable spectra. These data imply that the quenched and unquenched components of the emission emanate from the same excited state rather than two energetically distinct states. Photoluminescence lifetime measurements under oxygen-free conditions provided further evidence that emission in Zr(bppda)_2_ proceeds via two distinct pathways occurring on vastly different time scales. A long-lived component was readily modeled using a simple single-exponential decay with a lifetime of τ = 294 μs (Figure 3, bottom). This slow emission process is strongly quenched by ^3^O_2_ and therefore completely suppressed under aerobic conditions. The second, short-lived component can be clearly identified by the rapid loss of emission intensity prior to the onset of the long-lived decay under oxygen-free conditions (Figure 3, bottom). This fast emission process is retained in the presence of ^3^O_2_ and exhibits a lifetime that is shorter than the 1 ns time resolution of our time-correlated single photon counting (TCSPC) setup.

Taken together, the experimental observations for the photoluminescence of Zr(bppda)_2_ at room temperature are consistent with a combination of prompt fluorescence, that is not affected by ^3^O_2_ due to its short lifetime, and long-lived thermally activated delayed fluorescence, TADF, involving a long-lived triplet excited state that is effectively quenched by ^3^O_2_. Most importantly, the identical emission profiles in the presence and absence of ^3^O_2_ strongly disfavor an alternative interpretation involving direct triplet state deactivation via phosphorescence, but instead suggest that both short- and long-lived emission emanate from the same singlet excited state. The simultaneous observation of short- and long-lived emission and the partial quenching by ^3^O_2_ requires that direct singlet-state deactivation to the ground state via prompt fluorescence or nonradiative processes and intersystem crossing to a long-lived triplet state are competitive processes and must occur at comparable rates.

To investigate the potential influence of solvent polarity on the absorption and emission profiles, electronic absorption and emission spectra were recorded in a total of four solvents covering a wide range of polarities (benzene, THF, dichloromethane, and dimethyl sulfoxide). The near identical spectra (Figure S7 and S8) indicate the absence of any significant solvatochromism and suggest that the electronic dipole moment of Zr(bppda)_2_ does not change significantly upon excitation. Considering the *D*_2*d*_ symmetry of the molecule in solution established by NMR spectroscopy, no dipole moment can be present in the electronic ground state. Consequently, the lowest energy excited state must also lack a dipole moment, which suggests that it must be delocalized over both bppda^2–^ ligands. Note that metal-centered excited states that typically show only small changes in dipole moment can be excluded for a Zr^IV^ complex with a d^0^ electron configuration and would also be inconsistent with the high extinction coefficient for the lowest energy absorption band in Zr(bppda)_2_. The lack of solvatochromism in Zr(bppda)_2_ mirrors the spectroscopic behavior of closely related Zr(PDP)_2_ complexes, while the proposed delocalized nature of the excited state is supported computationally (*vide infra*).

### Temperature-Dependent Emission Studies

**Figure 4:**
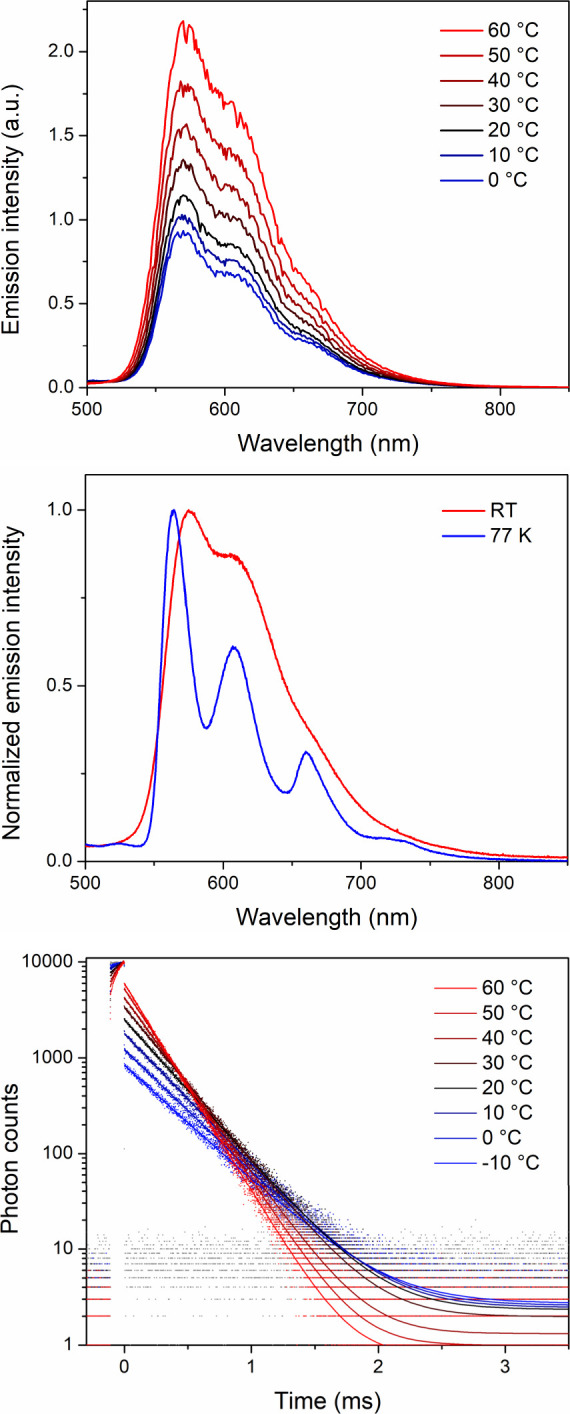
Top: Temperature-dependent emission spectra for Zr(bppda)_2_ recorded in THF solution under a dinitrogen atmosphere (λ_ex_ = 480 nm). Middle: Comparison of the emission profiles of Zr(bppda)_2_ in 2-MeTHF at room temperature and in frozen solution at 77 K. Bottom: Temperature-dependent photoluminescence decay traces (570 nm detection) recorded in THF solution under a dinitrogen atmosphere following excitation at 516 nm. Solid lines represent single-exponential fits of the variable-temperature data. The instrument response function is shown in gray.

To further support our TADF hypothesis, temperature-dependent emission studies were conducted. Consistent with emission by TADF, the photoluminescence characteristics of Zr(bppda)_2_ are highly sensitive to changes in temperature. Emission profiles in THF solution recorded on the same sample between 0 and 60 °C are shown in the top section of Figure 4 and clearly show an increase in emission intensity with increasing temperature. This temperature dependence unambiguously demonstrates the thermally activated nature of a significant component of the observed photoluminescence and is characteristic for systems with emission by TADF.^54^ In contrast, long-lived emission by a phosphorescence mechanism typically displays decreasing emission intensities upon increasing temperature due to more facile nonradiative decay at elevated temperatures. As expected for a TADF system, the lifetime of the long-lived component of the emission is also highly sensitive to temperature and declines steadily with increasing temperature from τ_TADF_ = 357 μs at −10 °C to τ_TADF_ = 198 μs at 60 °C (Figure 4, bottom).

To further investigate any potential emission processes in the absence of TADF, a frozen solution emission spectrum was obtained by cooling a 2-MeTHF solution of Zr(bppda)_2_ to 77 K (Figure 4, middle). Due to the small amount of available thermal energy under these cryogenic conditions, the TADF mechanisms is expected to be completely suppressed even in an anaerobic setting, which often allows the observation of phosphorescence in TADF systems and thereby provides a good estimate for the energy of the triplet state. However, a comparison of the emission spectra recorded for Zr(bppda)_2_ in liquid 2-MeTHF solution at room temperature and under frozen solution conditions at 77 K revealed only minor differences. The low-temperature spectrum is slightly blue-shifted and shows a more pronounced fine structure due to reduced line broadening compared to the solution data. These observations are expected for frozen-solution measurements and are the outcome of embedding the chromophore in a rigid matrix, resulting in a hypsochromic shift due to rigidochromism and reduced line broadening due to restriction of molecular motions. Three clearly resolved emission maxima can be observed at 564, 608, and 660 nm, and are consistent with a vibronic progression with ν = 1290 cm^–1^. An additional, poorly resolved weak feature can be detected around 723 nm. More interestingly, the lack of any clear new features in the low temperature data resulting from phosphorescence implies that the triplet state of Zr(bppda)_2_ is either completely nonemissive or exhibits a very low phosphorescence quantum yield that prevents its detection in the presence of efficient prompt fluorescence at low temperature. This is in stark contrast to previously reported zirconium TADF emitters such as Zr(^Mes^PDP^Ph^)_2_, which shows exclusively phosphorescence at 77 K due to facile intersystem crossing,^11^ and the related main group compounds E(^Me^PDP^Ph^)_2_ (E = Si, Ge, Sn) that exhibit dual emission under cryogenic conditions due to prompt fluorescence and phosphorescence.^55^

### Transient Absorption Spectroscopy

**Figure 5:**
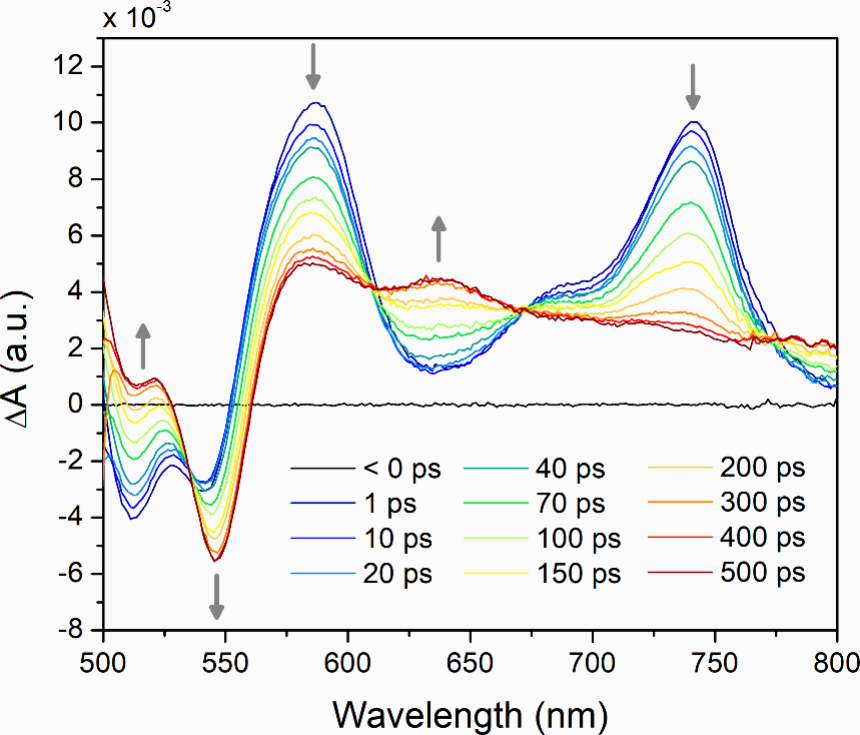
Time-resolved transient difference spectra recorded at selected delay times after pulsed laser excitation at 480 nm. The transient difference spectrum of the T_1_ state, shown in red, persists over the full delay time of the TA experiment (7 ns).

The excited-state dynamics of Zr(bppda)_2_ after photoexcitation were probed by femtosecond transient absorption (fs-TA) spectroscopy conducted in THF solution at room temperature. The transient difference spectra obtained following pulsed excitation at 480 nm are shown in Figure 5. At short delay times, several characteristic features are observed in the spectral region between 500 and 800 nm, which can be assigned as two ground state bleaches with minima at 511 and 539 nm and two excited state absorption bands with maxima at 586 and 740 nm. A third excited state absorption feature is visible as a shoulder around 685 nm. This spectral signature was assigned to the S_1_ excited state of Zr(bppda)_2_ formed immediately upon excitation. Within 500 ps, the S_1_ state converts to a long-lived excited state that persists over the entire delay time of the fs-TA experiment (7 ns) and, therefore, was assigned as the T_1_ excited state. The transient difference spectrum of this state shows a ground state bleach with a maximum at 545 nm and several broad T_1_ → T_n_ excited state absorption features ranging from 561 nm to the limit of the recorded spectral window at 800 nm with shallow maxima at 582 and 638 nm. Considering the information from steady-state and time-resolved emission studies that clearly established emission through a combination of prompt and delayed fluorescence, a three-state model including direct S_1_ → S_0_ deactivation (radiative and nonradiative) and facile S_1_ → T_1_ intersystem crossing was employed to fit the data. This simplified kinetic approach assumes that reverse intersystem crossing, T_1_ → S_1_, and direct triplet state deactivation, T_1_ → S_0_ are slow compared to initial S_1_ decay, which is reasonable considering the long lifetime of the TADF emission. Note that the proposed three-state model is also consistent with the observation of well-defined isosbestic points at 611, 671, and 774 nm despite the involvement of three distinct species (S_1_, T_1_, and S_0_) as demonstrated by Han and Spangler.^56^ Global analysis of the TA spectroscopic data yielded a time constant of 142 ps for initial S_1_ deactivation and population of the T_1_ state (Figure S10), which is consistent with the subnanosecond lifetime of the prompt fluorescence observed by TCSPC. While this time constant reflects a composite of multiple kinetic processes resulting in S_1_ deactivation and should not be mistaken for the time constant of intersystem crossing, τ_ISC_, that can be extracted from similar TA spectroscopic measurements in systems without prompt fluorescence, it provides a lower boundary for τ_ISC_ in Zr(bppda)_2_.

### Density Functional Theory

To gain further insight into the electronic structure and the nature of the observed optical transitions for Zr(bppda)_2_, density functional theory (DFT) calculations were conducted at the B3LYP level. The ground-state geometry was optimized as a closed-shell singlet using the untruncated molecular structure obtained by X-ray diffraction as the starting point. To account for the *D*_2*d*_ symmetric molecular structure determined by NMR spectroscopy in solution, symmetry constraints were imposed during geometry refinement using the automatic point group detection algorithm included in the ORCA 5.0 program suite.^57,58^ Important structural parameters are summarized in Table 1 and are in excellent agreement with the experimental values.

**Figure 6:**
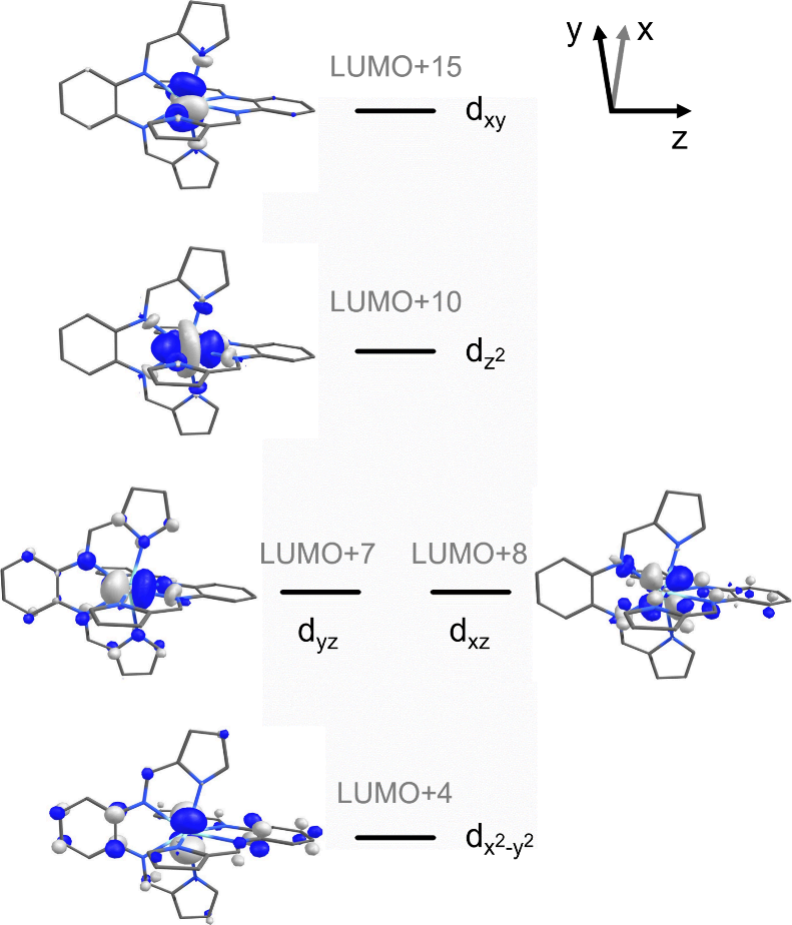
Qualitative d-orbital splitting diagram showing the interactions between the bppda^2–^ ligands and the Zr 4d orbitals.

As expected, all molecular orbitals with significant contributions (>20%) from the zirconium 4d orbitals are unoccupied, confirming the assignment of Zr(bppda)_2_ as a Zr^IV^ species. All occupied frontier molecular orbitals (HOMO to HOMO–6) are exclusively ligand centered with Zr contributions below 1%. The HOMO and HOMO–1 are degenerate and contain major contributions from the pyrrolide and phenylenediamine π-systems. Considering the less common eight-coordinate ligand field around the zirconium center, it is instructive to examine the interactions of each d-orbital with the two bppda^2–^ ligands. Using the coordinate system of the *D*_2*d*_ point group as the reference frame, the *S*_4_ axis bisecting the two phenylenediamine units of Zr(bppda)_2_ was chosen as the *z* axis, while the x and y axes coincide with the perpendicular *C*_2_ axes passing between the two ligand planes. In this coordinate system, the d_*xy*_ (b_2_, LUMO+15) and d_z2_ (a_1_, LUMO+10) orbitals are most strongly destabilized by metal ligand interactions and are exclusively σ-antibonding (σ*) in character. Due to the off-axis alignment of the phenylenediamine nitrogen donors, the destabilization of the d_z2_ orbital is less than that of the d_*xy*_ orbital. As required by the molecular symmetry, the d_*xz*_ and d_*yz*_ orbitals are degenerate (e, LUMO+7 and LUMO+8) and can each be described as σ* with respect to the phenylenediamine unit of one ligand and π-antibonding (π*) with respect to the second one. The d_x2-y2_ orbital (b_1_) experiences the weakest interactions with the ligands and is best described as having minor π* character due to interactions with the four pyrrolide π-systems in the xy-plane. Notably, several unoccupied molecular orbitals exhibit significant Zr d_x2-y2_ contributions including most importantly the LUMO (25%), LUMO+4 (37%), LUMO+11 (12%). A qualitative d-orbital splitting diagram depicting the molecular orbitals with the highest contribution for each individual Zr 4d orbital is shown in Figure 6.

**Figure 7:**
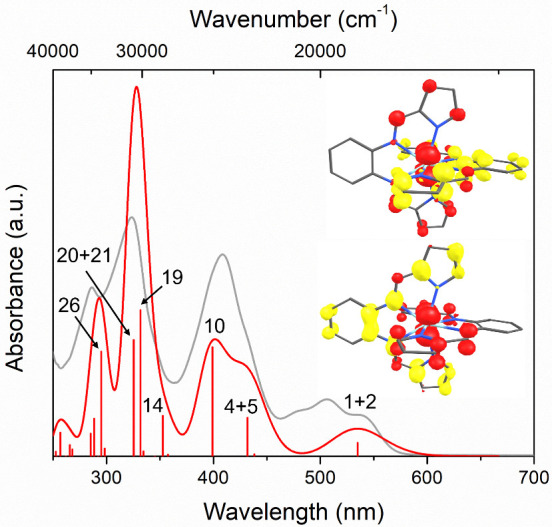
Comparison of the calculated (red) and experimental (gray) electronic absorption spectra of Zr(bppda)_2_ in THF. The stick plot indicates the positions and relative intensities of individual transitions predicted by TD-DFT. The most intense transitions are labeled according to their TD-DFT state number. The major contributions of individual single-electron excitations to each numbered state are listed in Table S2. The insets show the difference densities for the degenerate lowest energy states labeled as 1 + 2 and highlight the mixed ^1^IL/^1^LMCT character of the lowest energy absorption band (red: increased electron density; yellow: decreased electron density).

Time-dependent density functional theory (TD-DFT) calculations were performed to analyze the optical transitions observed in the electronic absorption spectrum of Zr(bppda)_2_. Solvent effects resulting from THF were modeled using the conductor-like polarizable continuum model (C-PCM) to mimic the experimental conditions.^59^ The calculated absorption spectrum is shown in Figure 7, and the most intense TD-DFT states (predicted oscillator strength *f*_osc_ > 0.05) are labeled according to their state number. Note that the TD-DFT module implemented in the ORCA 5.0 program suite does not consider molecular symmetry. As a result, degenerate excited states appear as multiple TD-DFT states with identical energies. The transition energies and contributing single-electron excitations (including their weight) for each TD-DFT state are given in Table S2. The computational data are in near perfect agreement with the experimental spectrum and reproduce all major absorption bands, including the feature visible only as a shoulder at 431 nm. The one qualitative difference between the experimental and calculated spectra is the lack of fine structure for the lowest energy absorption band in the computational data. This further supports the assignment of these features as a vibronic progression, which cannot be captured by a simple TD-DFT approach using a static geometry but would require more extensive computational approaches that are beyond the scope of this study.^60^

**Figure 8:**
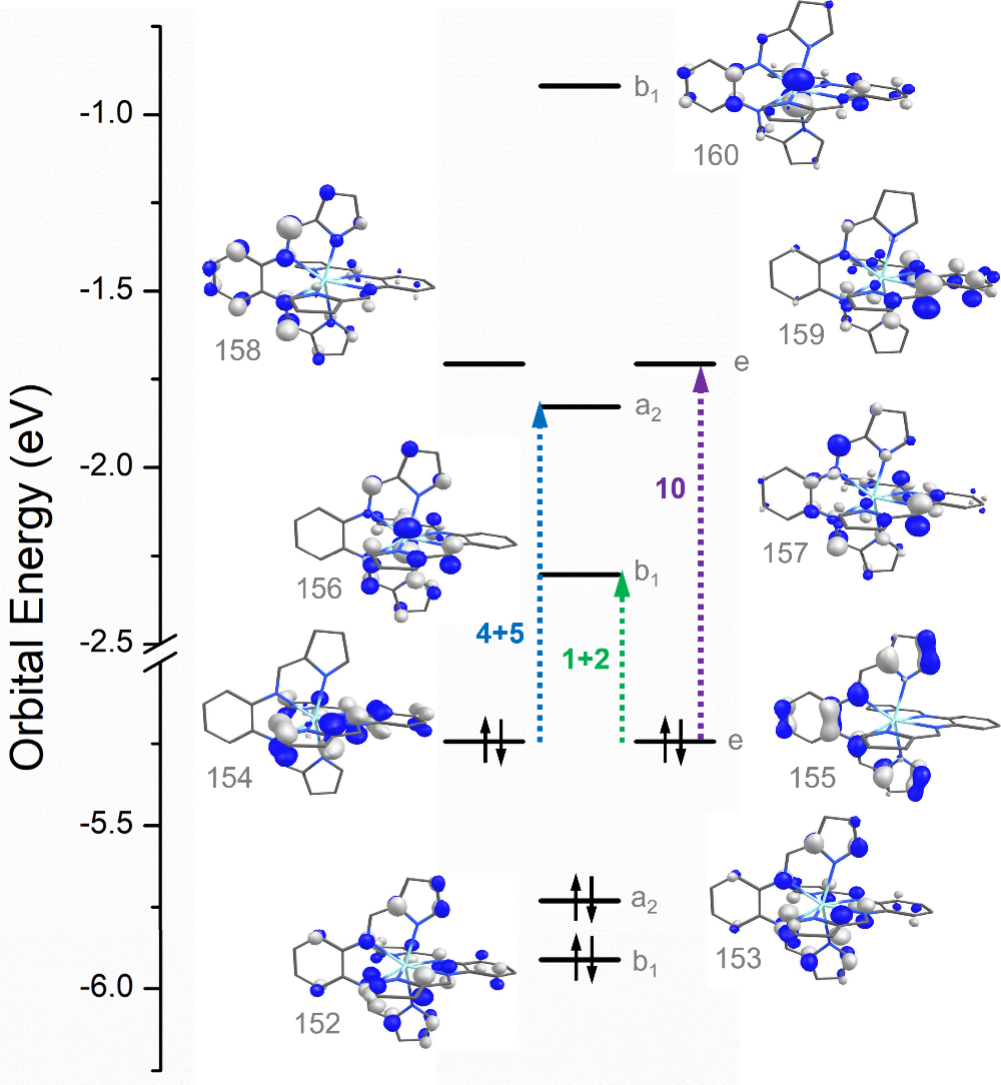
Frontier molecular orbital diagram of Zr(bppda)_2_ obtained from TD-DFT calculations. Symmetry labels according to the *D*_2*d*_ point group are provided in gray. Dotted vertical arrows indicate the dominant single-electron excitations corresponding to the transitions in the visible region of the calculated electronic absorption spectrum.

A more in-depth analysis of the individual excited states provided further insight into the nature of each electronic transition. A visualization of the frontier molecular orbital manifold, including simplified depictions of the main excitations leading to absorption of visible light, is shown in Figure 8. The lowest energy absorption band at 535 nm was computed to be the result of a degenerate singlet excited state (^1^E) corresponding to two degenerate single-electron excitations from the HOMO/HOMO–1 set to the LUMO (TD-DFT states 1 and 2), which are dipole allowed under *D*_2*d*_ symmetry. Based on the composition of the donor (0% Zr character) and acceptor orbitals (25% Zr character) this transition is best described as an intraligand (^1^IL) transition with significant ligand-to-metal charge transfer (^1^LMCT) contributions of 25%. A Mulliken population analysis of the unrelaxed densities for TD-DFT states 1 and 2 provided an alternative quantification method for the ^1^LMCT character of the lowest energy absorption band and showed substantial negative charge migration from the ligands to zirconium compared to the ground state (Δ*q*_Zr_ = −0.31 e), implying a slightly higher ^1^LMCT character of 31%. A visualization of this charge migration is provided in the unrelaxed difference densities between the ground state and the degenerate TD-DFT states 1 and 2 shown in in Figure 7.

The second absorption band in the visible part of the spectrum is the result of two energetically close lying excited states with computed absorption maxima at 432 and 399 nm. The lower energy feature, visible as a shoulder in the experimental and calculated spectra, is the result of a dipole-allowed, degenerate transition between the HOMO/HOMO–1 orbitals and the LUMO+1 (TD-DFT states 4 and 5). Because both donor and acceptor orbitals are exclusively ligand centered (0% Zr character), this ^1^E excited state is the result of a pure ^1^IL transition. This was also confirmed by Mulliken population analysis, which revealed negligible charge transfer involving the zirconium center (Δ*q*_Zr_ = −0.01 e). The higher energy transition, establishing the maximum of the absorption band around 400 nm, is the result of a single-electron excitation from the HOMO/HOMO–1 set to the degenerate LUMO+2/LUMO+3 pair. Note that out of the four possible excitations only one is dipole allowed under *D*_2*d*_ symmetry (TD-DFT state 10). Considering the very small amount of metal contribution to the acceptor orbitals (3% Zr character), this excited state can be described as the result of a ^1^IL transition with only minor charge migration to zirconium (Δ*q* = −0.11 e).

**Figure 9:**
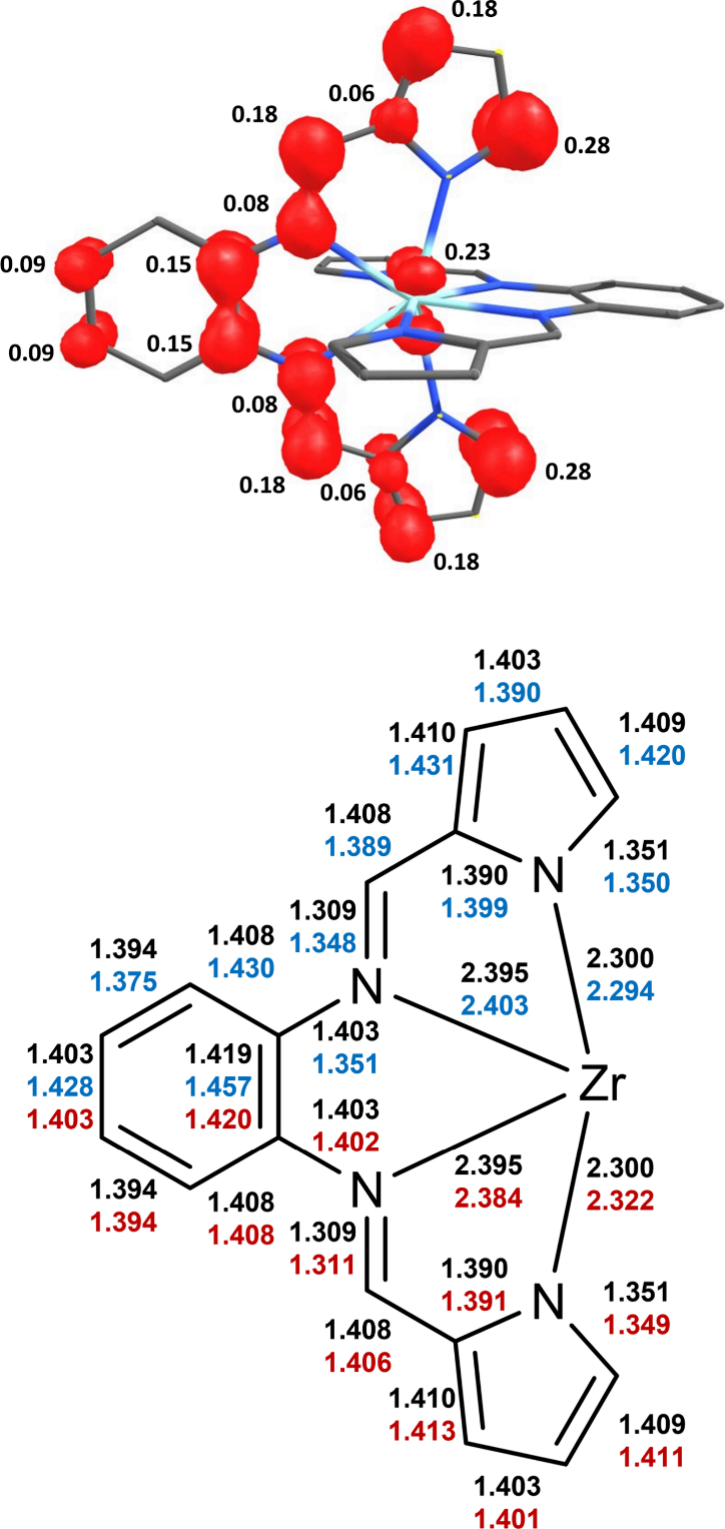
Top: Spin density plot for the lowest-energy triplet Zr(bppda)_2_ obtained from a Mulliken population analysis. Bottom: Comparison of the structural parameters between the singlet ground state (black) and lowest energy-triplet excited state of Zr(bppda)_2_ (blue and red) obtained by DFT geometry optimization. For the triplet state, the metrics for the two distinct ligands are color-coded. Due to the *C*_2*v*_ symmetry of the system, the upper and lower halves for each ligand are identical.

The electronic structure of the lowest energy triplet state was also examined by DFT calculations. Starting from the *D*_2*d*_ symmetric singlet state structure, a geometry optimization imposing no symmetry constraints yielded a *C*_2*v*_ symmetric geometry for the triplet state. This can be understood as the result of a Jahn–Teller distortion following promotion of an electron from the degenerate HOMO/HOMO–1 pair to the LUMO to allow a parallel allignment of the two electron spins in two SOMOs in the triplet state. The spin density plot shown in Figure 9 (top) supports that the lowest energy triplet state can in fact be described in this way because the spin distribution in the triplet closely resembles a simple superposition of the HOMO and LUMO orbitals of the singlet ground state shown in Figure 8. The spin density value of 0.23 for the central zirconium ion is also consistent with the 25% Zr character of the ground state LUMO turned SOMO for the triplet state and indicates similar LMCT contributions for the triplet state as in the lowest energy singlet state.

The geometric changes between the triplet excited and singlet ground state, resulting in a reduction of symmetry and lifting of degeneracy for the two bppda^2–^ units, are mostly confined to one of the ligands and are illustrated in Figure 9. The most prominent alterations are a lengthening of the two C = N bonds of the imines, a shortening of the N–C_Ph_ bonds, and an increase in the phenylene C–C bond. In addition, a noticeable quinoidal distortion of the phenylene unit with two short and four long C–C bonds is observed. These geometric changes are consistent with promotion of one electron from the HOMO to the LUMO of the singlet ground state.

### Comparison of Zr(bppda)_2_ and Zr(PDP)_2_ Luminophores

It is informative to compare the (electronic) structures and photophysical properties of Zr(bppda)_2_ with previously reported Zr(PDP)_2_ photosensitizers, which appear similar at first glance. Both types of complexes contain two polydentate ligands with fully conjugated, planar π-systems that are arranged in a perpendicular fashion to each other through coordination to the central zirconium ion, resulting in an overall *D*_2*d*_ symmetric molecular structure. The design of both ligand systems places the four electron-rich pyrrolide moieties in the xy-plane of the coordination sphere and forces the plane of the five-membered heterocycles to be aligned along the *z*-direction. Consequently, all π-interactions between the metal and the pyrrolide π-systems are constrained to the d_x2-y2_ orbital in both types of complexes. The most obvious structural difference between Zr(bppda)_2_ and Zr(PDP)_2_ are the bridging phenylenediamine and pyridine units, respectively, which connect the flanking pyrrolide moieties in each ligand. Even though both bridging fragments by themselves are formally charge neutral and best considered as mild π-acceptor ligands, conjugation between the pyrrolides and the phenylenediamine or pyridine subunit confers some amide nitrogen character to the central donor atoms of both ligand systems.

The switch from a monodentate pyridine bridging unit in PDP^2–^ to a bidentate phenylenedamine fragment in bppda^2–^ substantially changes the electronic structure of the zirconium center. For Zr(PDP)_2_, the pyridine units engage in strong σ-interactions with the d_z2_ orbital (σ*) as expected in a distorted octahedral structure. In addition, they can engage in π-interactions with the d_*xz*_ and d_*yz*_ orbitals. As a result, the LUMO of Zr(PDP)_2_ complexes is a degenerate set of orbitals with dominant contributions from the pyridine heterocycles and the Zr d_*xz*_ and d_*yz*_ orbitals. As described in the computational section (*vide supra*), a slightly different situation is encountered in Zr(bppda)_2_, where the phenylenediamine fragments engage in weaker σ-interactions with the d_z2_ orbital due to their off-axis position, but can form both σ- and π-interactions with the degenerate d_*xz*_/d_*yz*_ orbitals. This results in a stronger destabilization of the latter two d-orbitals and a nondegenerate LUMO with predominantly pyrrolide and Zr d_x2-y2_ character. Additionally, the switch from a pyridine to a phenylenediamine bridge changes the ordering of the occupied ligand donor orbitals in Zr(bppda)_2_ compared to Zr(PDP)_2_. For both both complexes, the HOMO to HOMO–3 orbitals (a_2_, b_2_, and e in *D*_2*d*_ symmetry) are closely related with major contributions from the pyrrolide π-system and nodal planes passing through each of the pyrrolide nitrogens. However, the e set constitutes the degenerate HOMO/HOMO–1 for Zr(bppda)_2_, while the a_2_ and b_2_ orbitals are the nondegenerate HOMO and HOMO–1 in Zr(PDP)_2_.

**Figure 10:**
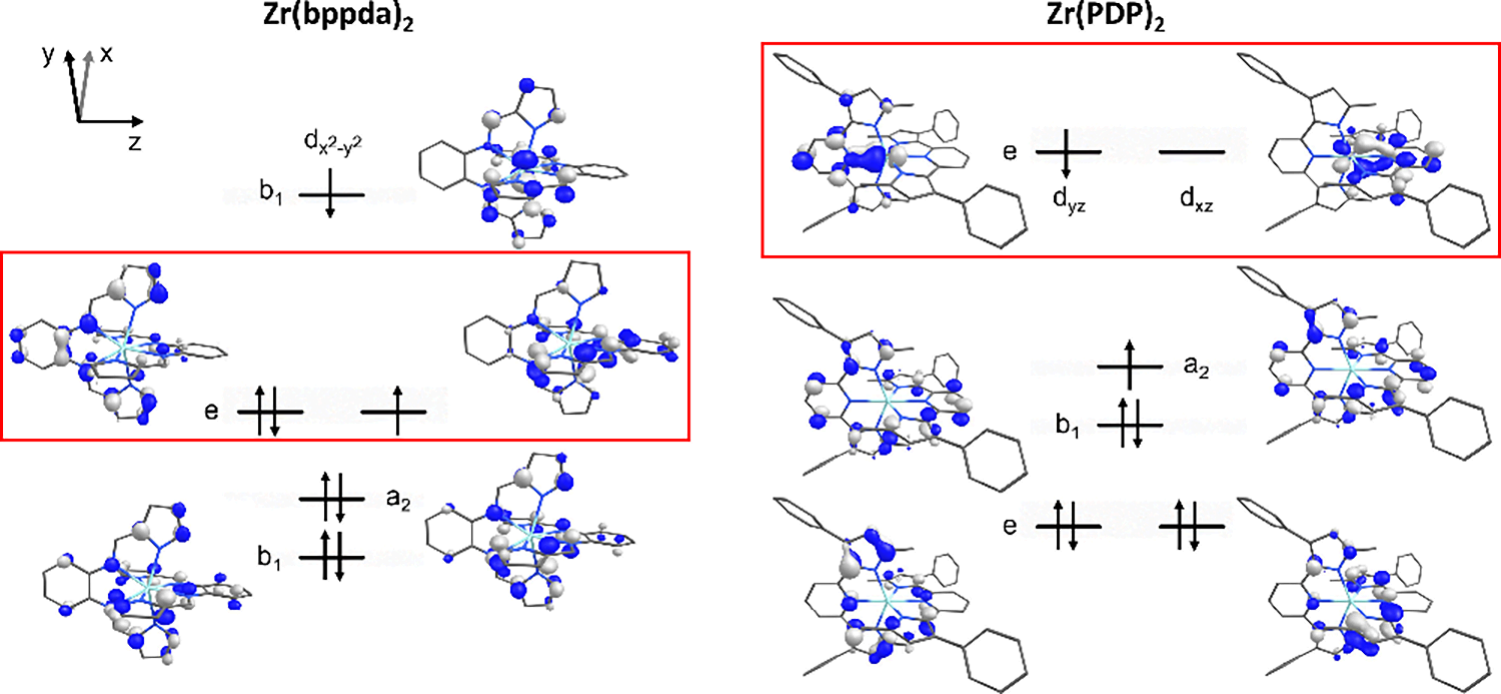
Comparison of the frontier molecular orbitals for Zr(bppda)_2_ (left) and Zr(PDP)_2_ (right), showing the lowest-energy electron configurations after single-electron excitation. The red boxes highlight the origin of the excited state degeneracy, which involves exclusively ligand-centered orbitals for Zr(bppda)_2_ and orbitals with significant Zr contributions for Zr(PDP)_2_.

Overall, the changes in both the HOMO and LUMO result in different frontier molecular orbital manifolds with a nondegenerate HOMO and degenerate LUMO/LUMO+1 with d_*xz*_/d_*yz*_ character for Zr(PDP)_2_ but a degenerate HOMO and nondegenerate LUMO with d_x2-y2_ character for Zr(bppda)_2_ (Figure 10). We propose that this subtle change in electronic structure is reflected in the photophysical properties of Zr(bppda)_2_ and Zr(PDP)_2_. Both types of complexes show strong visible-light absorption due to spin- and dipole-allowed HOMO/LUMO transitions with ^1^IL/^1^LMCT character. In each system, these transitions are degenerate, resulting in a Jahn–Teller distortion of the excited state geometry. Following intersystem crossing (ISC) to a long-lived triplet state with ^3^IL/^3^LMCT character, long-lived emission proceeds by a TADF mechanism with lifetimes of hundreds of microseconds at room temperature. The most important difference between the two systems is the efficiency and rate of ISC. This process is rapid in Zr(PDP)_2_ complexes (τ_ISC_ = 12 ps for Zr(^Mes^PDP^Ph^)_2_), resulting in efficient population of the triplet state (Φ_ISC_ ≈ 100%) and no detectable prompt fluorescence.^11^ In contrast, the analogous process in Zr(bppda)_2_ is at least an order of magnitude slower than in Zr(PDP)_2_ as established by TA spectroscopy (*vide supra*) and occurs on a similar time scale as prompt fluorescence, resulting in incomplete population of the triplet state (Φ_ISC_ < 100%) and emission on two very different time scales. While the lowest-energy singlet and triplet excited states are degenerate for both systems under the initial *D*_2*d*_ symmetry, we hypothesize that the origin of the degeneracy plays an important role in the ISC process. The degeneracy for Zr(PDP)_2_ is due to the LUMO/LUMO+1 set, which contains significant contributions from the zirconium center that possesses a large spin–orbit coupling constant and can facilitate efficient ISC. In contrast, the degeneracy for Zr(bppda)_2_ is the result of the HOMO/HOMO–1 set, which is exclusively ligand centered and contains no heavy atom contributions. The inefficiency of ISC in Zr(bppda)_2_ should also apply to reverse ISC from the triplet to the singlet manifold required for TADF emission and should result in longer residence times in the triplet state. More favorable nonradiative decay from the T_1_ state of Zr(bppda)_2_ under these conditions could help explain the poor photoluminescence quantum yield observed for this complex. However, it should be noted that the photoluminescence quantum yield in Zr(PDP)_2_ complexes is strongly dependent on the PDP substituents.^10^ Therefore, improvements in quantum yield for Zr(bppda)_2_ complexes through systematic substitution of the ligand framework may be possible in the future.

## Conclusions

In the present work, we reported the preparation and characterization of the novel photoluminescent zirconium complex Zr(bppda)_2_. The combination of two tetradentate, meridionally coordinating bppda^2–^ ligands with a Zr^IV^ center results in a *D*_2*d*_-symmetric, eight-coordinate complex that is remarkably stable toward hydrolysis and can be readily handled under regular benchtop conditions. Excitation at wavelengths below 550 nm resulted in photoluminescence, albeit with a low quantum yield of only 0.4%. A series of photophysical experiments including temperature-dependent emission spectroscopy and photoluminescence lifetime measurements under inert atmosphere and aerobic conditions established that the emission in Zr(bppda)_2_ can be attributed to a combination of prompt fluorescence (τ_PF_ < 1 ns) and thermally activated delayed fluorescence (TADF, τ_TADF_ = 294 μs) at room temperature. This description was further supported by transient absorption spectroscopy, which established the involvement of a long-lived triplet excited state and provided a lifetime of 142 ps for the lowest-energy singlet excited state. Further analysis by TD-DFT calculations established moderate yet significant LMCT character of 25–30% for the lowest energy singlet and triplet excited states. Taken together, our findings demonstrate that Zr(bppda)_2_ represents a new addition to the growing field of group 4 chromophores that rely on metal d-orbital contributions to the acceptor orbitals of the initial charge transfer step, imparting LMCT character to their emissive states. Additional insight was gained by comparison of Zr(bppda)_2_ with closely related Zr(PDP)_2_ complexes. Despite their structural similarities with *D*_2*d*_-symmetric geometries incorporating four pyrrolide moieties, the change from a bridging pyridine unit in Zr(PDP)_2_ to phenylenediamine in Zr(bppda)_2_ and the associated shift from a six-coordinate to an eight-coordinate Zr^IV^ center lead to subtle changes in the electronic structure that are reflected in the photophysical properties. Most importantly, the rate of intersystem crossing is at least 1 order of magnitude slower for Zr(bppda)_2_, which we propose to be due to the removal of degeneracy for the LUMO and LUMO+1. These orbitals exhibit significant Zr d-orbital contributions in both Zr(PDP)_2_ and Zr(bppda)_2_ complexes, but only allow for the generation of large orbital angular momentum contributions and strong spin–orbit coupling in degenerate or near-degenerate configurations. This hypothesis could have important implications for the design of new photoluminescent early transition metal complexes in the future.

## Experimental Details

### General Considerations

All air- and moisture-sensitive manipulations were carried out using standard high vacuum line, Schlenk, or cannula techniques or in an MBraun inert atmosphere drybox containing an atmosphere of purified nitrogen. Solvents for air- and moisture-sensitive manipulations were dried and deoxygenated using a Glass Contour Solvent Purification System and stored over 4 Å molecular sieves. All solids were dried under high vacuum overnight in order to bring them into the glovebox. Benzene-*d*_6_ for NMR spectroscopy was distilled from sodium metal. Tetrabenzylzirconium (ZrBn_4_)^61^ and *N*,*N*′-bis(2-pyrrylmethylidene)-1,2-phenylenediamine (H_2_bppda)^53^ were prepared as reported previously. All remaining chemicals were purchased from commercial sources and used as received.

### Preparation of Zr(bppda)_2_

A solution of tetrabenzyl zirconium (130 mg, 0.286 mmol, 0.50 equiv) in 2 mL of benzene was added slowly to a 20 mL vial charged with a solution of H_2_bppda (150 mg, 0.572 mmol, 1.00 equiv) in 3 mL of benzene. A dark orange precipitate formed upon stirring the reaction for 30 min at room temperature. The resulting suspension was then filtered and the solid was washed three times with cold Et_2_O. The product was collected as a dark orange microcrystalline solid (Yield: 164 mg, 94%). ^1^H NMR (400 MHz, C_6_D_6_; δ, ppm): 7.94 (s, 4H, N = C*H*), 7.12 (s, 4H, Pyrrole*H*), 7.04–6.94 (m, 8H, Ph*H*), 6.47 (m, 4H, Pyrrole*H*), 6.04 (m, 4H, Pyrrole*H*). ^13^C{^1^H} NMR (101 MHz, C_6_D_6_; δ, ppm): 149.3, 142.9, 142.3, 140.4, 126.3, 121.8, 115.8, 113.8. Anal. Calcd for C_26_H_18_N_6_Zr: C, 62.82; H, 3.95; N, 18.32. Found: C, 62.97; H, 3.94; N, 18.42. Single crystals suitable for X-ray crystallographic analysis were grown from a saturated solution of Zr(bppda)_2_ in C_6_D_6_ at room temperature.

### X-ray Crystallography

A single crystal suitable for X-ray diffraction was coated with polyisobutylene oil (Sigma-Aldrich) in a drybox, mounted on a nylon loop, and then quickly transferred to the goniometer head of a Bruker AXS D8 Venture fixed-chi X-ray diffractometer equipped with a Triumph monochromator, a Mo Kα radiation source (λ = 0.71073 Å), and a PHOTON 100 CMOS detector. The sample was cooled to 100 K with an Oxford Cryostream 700 system and optically aligned. The APEX3 software program (version 2016.9–0) was used for diffractometer control, preliminary frame scans, indexing, orientation matrix calculations, least-squares refinement of cell parameters, and the data collection. Three sets of 12 frames each were collected using the omega scan method with a 10 s exposure time. Integration of these frames followed by reflection indexing and least-squares refinement produced a crystal orientation matrix for the crystal lattice that was used for the structural analysis. The data collection strategy was optimized for completeness and redundancy using the Bruker COSMO software suite. The space group was identified, and the data were processed using the Bruker SAINT+ program and corrected for absorption using SADABS. The structures were solved using direct methods (SHELXS) completed by subsequent Fourier synthesis and refined by full-matrix least-squares procedures using the programs provided by SHELXL-2014.

### Spectroscopic Measurements

^1^H and ^13^C {^1^H} NMR spectra were recorded on an Agilent 400 MHz DD2 spectrometer equipped with a 5 mm One NMR probe using NMR tubes fitted with J-Young valves. All chemical shifts are reported relative to SiMe_4_ using ^1^H (residual) chemical shifts of the solvent as a secondary standard. Optical spectroscopy experiments were performed in gastight quartz cuvettes with a 10 mm path length fitted with J-Young valves. Room-temperature electronic absorption spectra were recorded using a Shimadzu UV-1800 spectrophotometer. Room-temperature steady-state emission spectra were obtained using a Shimadzu RF-5301 PC spectrofluorophotometer. Steady-state emission spectra from 0 to 60 °C were recorded using a Horiba Jobin Yvon Fluorolog-3 Spectrofluorometer equipped with a 450 W Xe arc lamp as the excitation source and a Horiba FL-1073 photomultiplier tube (PMT). The same setup was used to determine temperature-dependent emission lifetimes using a single photon counting module in multichannel scaler mode and a 527 nm NanoLED pulsed excitation source. Emission lifetimes were determined using the provided decay analysis software package, DAS v6.1. Ultrafast TA measurements were conducted with a 1 kHz Libra, a Ti:sapphire regenerative amplifier system (Coherent Libra), which produces a ∼ 800 nm pulse with ∼45 fs temporal resolution with ∼4 W power. Using a beamsplitter, the output of the Libra was separated into pump and probe beam paths. The pump beam was directed to an optical parametric amplifier (Light Conversion OPerA). The optical parametric amplifier converts 800 nm Libra output into 480 nm to excite the IL/LMCT transition of Zr(bppda)_2_. The beams were directed to the commercial TA spectrometers. We used Helios (Ultrafast System) and EOS (Ultrafast systems) for fs- and μs-TA, respectively. A visible- light continuum, in the 400–800 nm spectral region, was generated by focusing onto a Ti:sapphire crystal. Optical filters were integrated in the probe beam path for rejection of the residual, unamplified, 800 nm radiation. TA measurements were conducted under the magic angle condition where polarization of the probe is 54.7° relative to the pump. Control of the pump and probe polarizations was achieved with two sets of λ/2 waveplate and polarizer combinations placed in both pump (before the sample) and probe (before continuum generation) beam paths.

### Computational Details

All calculations were performed using the ORCA quantum chemical program package v5.0.1.^57,58^ Geometry optimizations and TD-DFT calculations used the B3LYP density functional.^62^ In all cases, scalar-relativistic effects were included via the zeroth-order regular approximation (ZORA).^63^ The relativistically recontracted triple-ζ quality basis set, ZORA-def2-TZVP,^64^ was used for nitrogen atoms while the SARC-ZORA-TZVP was used for zirconium.^65^ All other atoms were handled with the recontracted split-valence ZORA-def2-SVP basis set.^64^ The calculations were accelerated using the RIJCOSX approximation in tandem with the decontracted SARC/J auxiliary basis set.^66,67^ All solvation effects resulting from tetrahydrofuran were handled using the conductor-like polarizable continuum model (C-PCM) and a Gaussian charge scheme.^59^ All molecular orbital and density plots were generated using Chemcraft.^68^

## Supporting Information Available

Additional experimental procedures, spectroscopic and crystallographic data, and computational details (PDF)
