# Supplementary material for: Photophysical Studies of a Zr(IV) Complex with Two Pyrrolide-Based Tetradentate Schiff Base Ligands
Source: Inorg Chem. 2024 May 3;63(20):9002–13. doi: 10.1021/acs.inorgchem.4c00365 (PMC11110004; doi:10.1021/acs.inorgchem.4c00365)
Supplement: Supplementary file 1 — ic4c00365_si_001.pdf [file ic4c00365_si_001.pdf]

Photophysical Studies of a Zr(IV) Complex with Two Pyrrolide-Based Tetradentate Schiff Base Ligands

Yu Zhang,[a,b,] Tia S. Lee,[c] Jeffrey L. Petersen,[a] and Carsten Milsmann[a,*]*

[a]C. Eugene Bennett Department of Chemistry, West Virginia University, Morgantown, West Virginia 26506, United States

[b]Department of Chemistry, Tufts University, Medford, Massachusetts 02144, United States

[c]Department of Chemistry, Princeton University, Princeton, New Jersey 08544, United States

yu.zhang@tufts.edu

camilsmann@mail.wvu.edu

Supporting Information

Contents

1. X-Ray Crystallography 3

2. NMR Spectroscopic Data 4

3. Optical Spectroscopy 6

3.1 Quantum Yield Determination 6

3.2 Additional Absorption and Emission Data 7

3.3 Transient Absorption Spectroscopy 9

4. Computational Studies 11

4.1 Input File Examples 11

4.2 Additional Results from TD-DFT Calculations 12

4.3 xyz-Coordinates 13

5. References 16

1. X-Ray Crystallography

Table S1. Crystallographic data for $Zr(bppda)_2 \cdot C_6H_6$.

	$Zr(bppda)_2 \cdot C_6H_6$
chem. formula	$C_{38}H_{30}N_8Zr$
cryst size, mm^3	0.266 x 0.345 x 0.472
Fw, g mol^{-1}	689.92
space group	P 3_221
a, Å	12.6811(5)
b, Å	12.6811(5)
c, Å	16.8875(7)
α, deg	90
β, deg	90
γ, deg	120
V, Å	2351.9(2)
Z	3
T, K	100(2)
ρ calcd, g cm^{-3}	1.461
reflns collected/$2\Theta_{max}$	47657/69.96
unique reflns/I > 2σ(*I*)	6901/6540
No. of params/restraints	214/0
λ, Å	0.71073
R1[a]/goodness of fit[b]	0.0234/1.060
wR2[c] (*I* > 2σ(*I*))	0.0565
Residual density, e$Å^{-3}$	+0.684/-0.408

[a]Observation criterion: *I* > 2σ(*I*), $R_1 = \sum((||F_O| - |F_C||) / \sum|F_O|$. [b]GoF = $[\sum[w(F_o^2 - F_c^2)^2] / (n-p)]^{1/2}$. [c]$wR_2 = [\sum[w(F_o^2 - F_c^2)^2] / \sum[w(F_o^2)^2]]^{1/2}$

2. NMR Spectroscopic Data

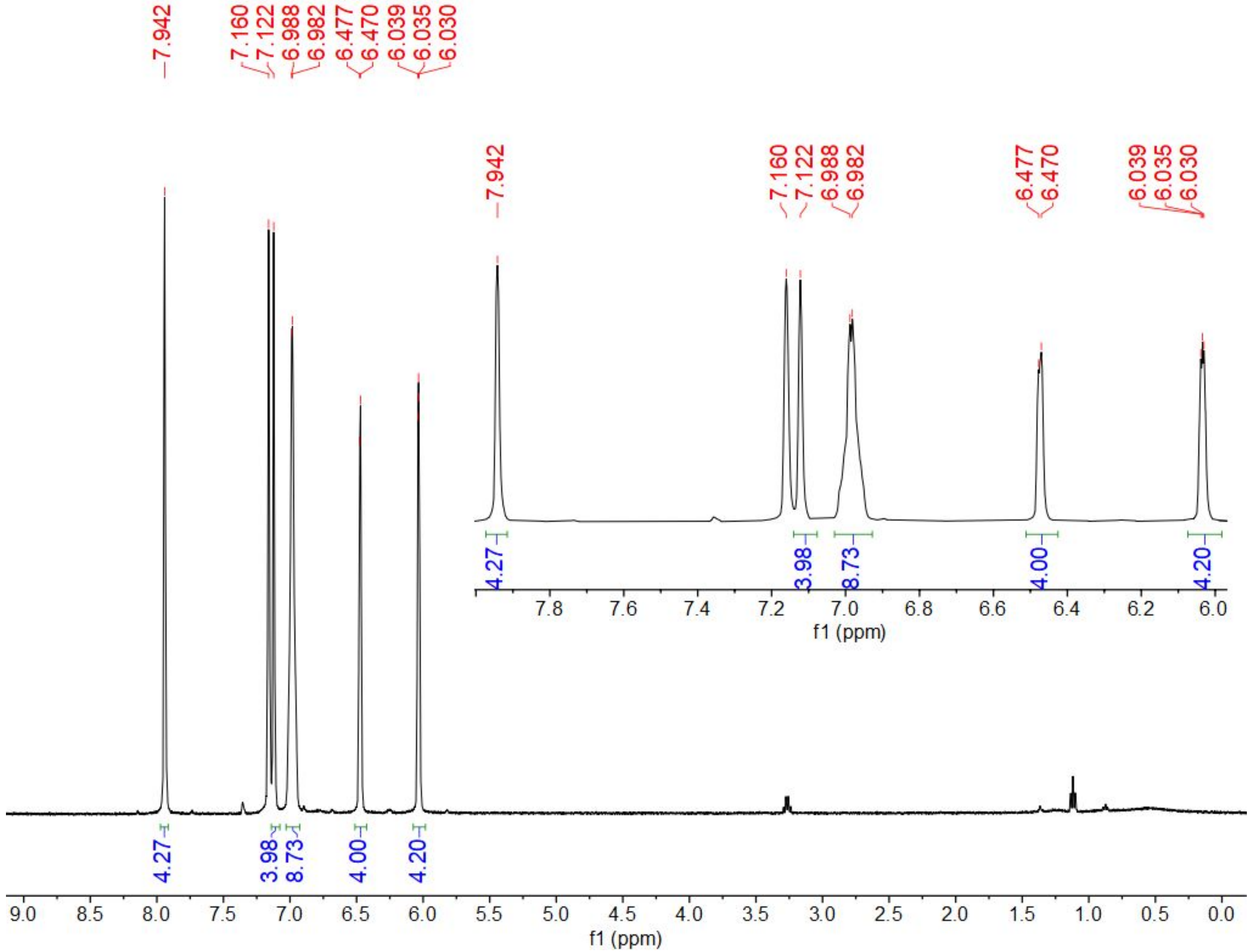

Figure S1. ^{1}H NMR spectrum (400 MHz) of Zr(bppda)$_2$ in benzene-d_6 at room temperature.

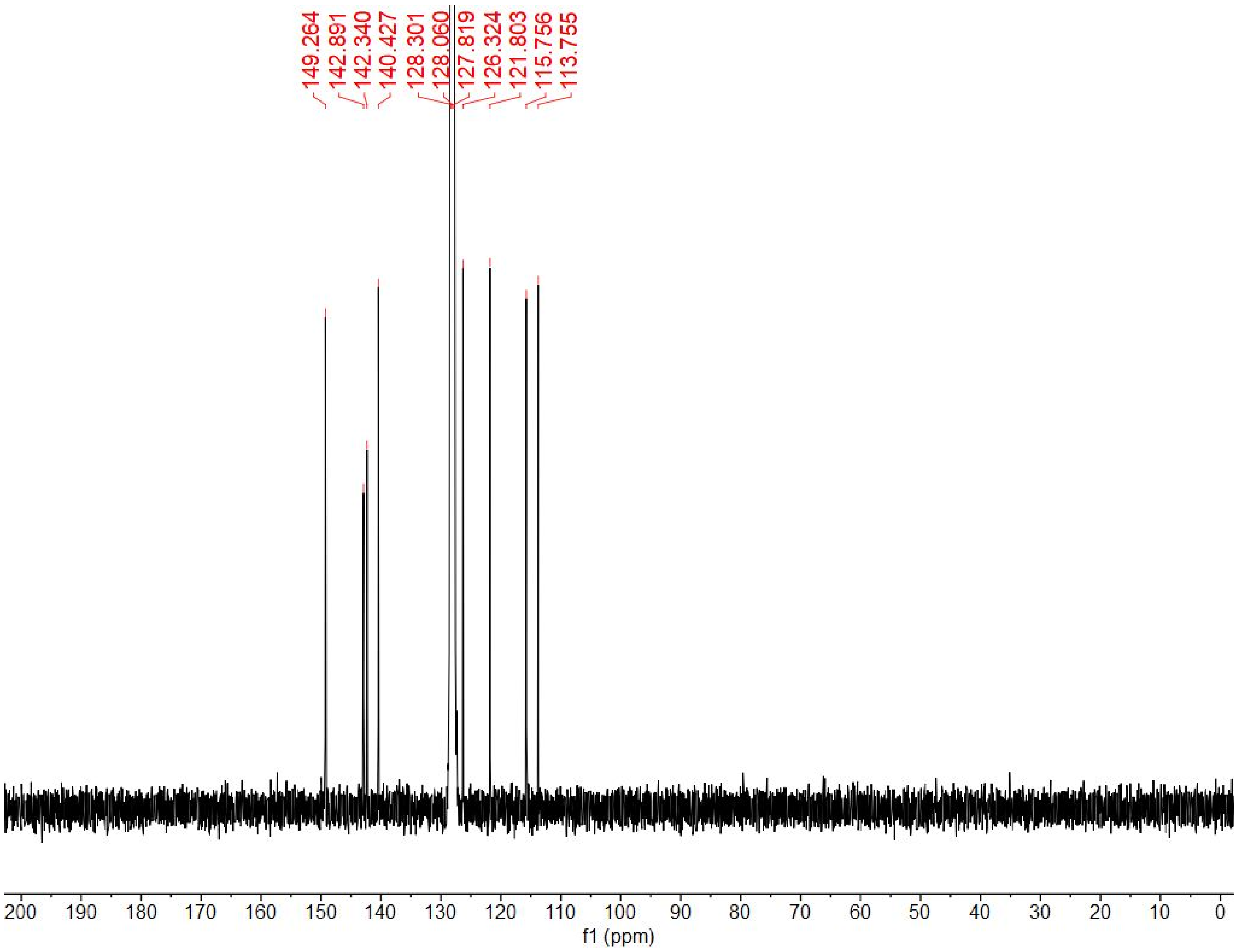

Figure S2. $^{13}C\{^1H\}$ NMR spectrum (100 MHz) of Zr(bppda)$_2$ in benzene-d_6 at room temperature.

3. Optical Spectroscopy

3.1 Quantum Yield Determination

The quantum yield of Zr(bppda)$_2$ was determined via comparative method.[1,2] Rhodamine 6G in ethanol (Φ = 0.94) was used as a standard and the excitation wavelength for all photoluminescence intensity measurements was kept constant at 488 nm.[3] The quantum yield was calculated by using the following equation:

$$\Phi_X = \Phi_{ST}\left(\frac{Grad_X}{Grad_{ST}}\right)\left(\frac{n_X^2}{n_{ST}^2}\right)$$

The subscripts *X* and *ST* stand for the sample and the standard, respectively. Φ is the photoluminescence quantum yield. *Grad* is the gradient from the plot of integrated photoluminescence intensity versus absorbance, and *n* is the refractive index of the solvent.

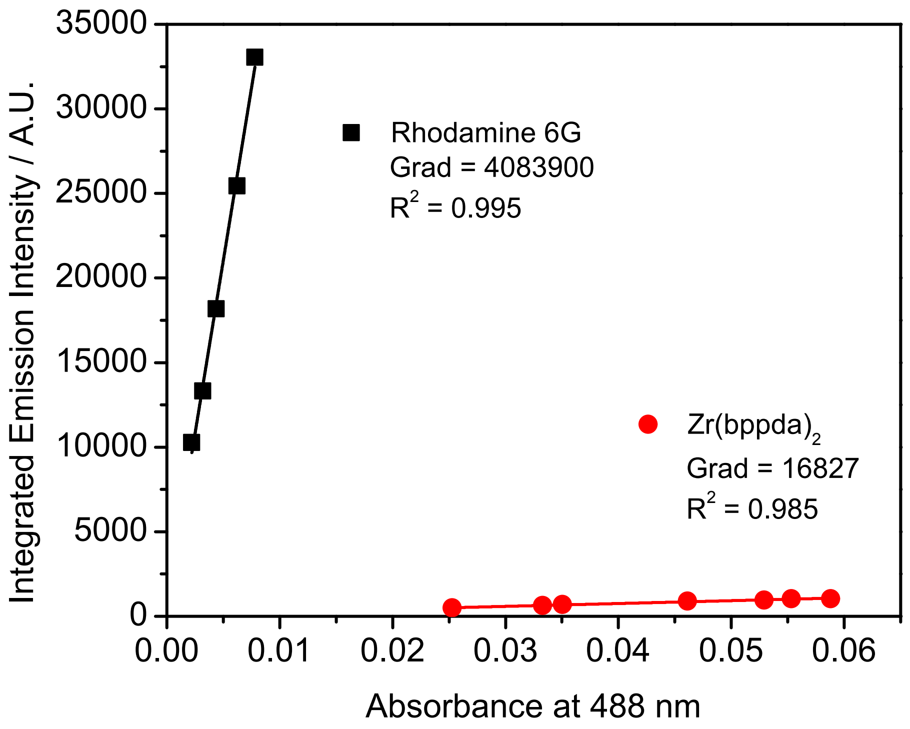

Figure S3. Plot of absorbance at 488 nm versus integration of the emission spectrum for Zr$^{(bppda)}$$_2$ in THF and the standard, rhodamine 6G, in ethanol. The solid lines represent linear fits of the data.

3.2 Additional Absorption and Emission Data

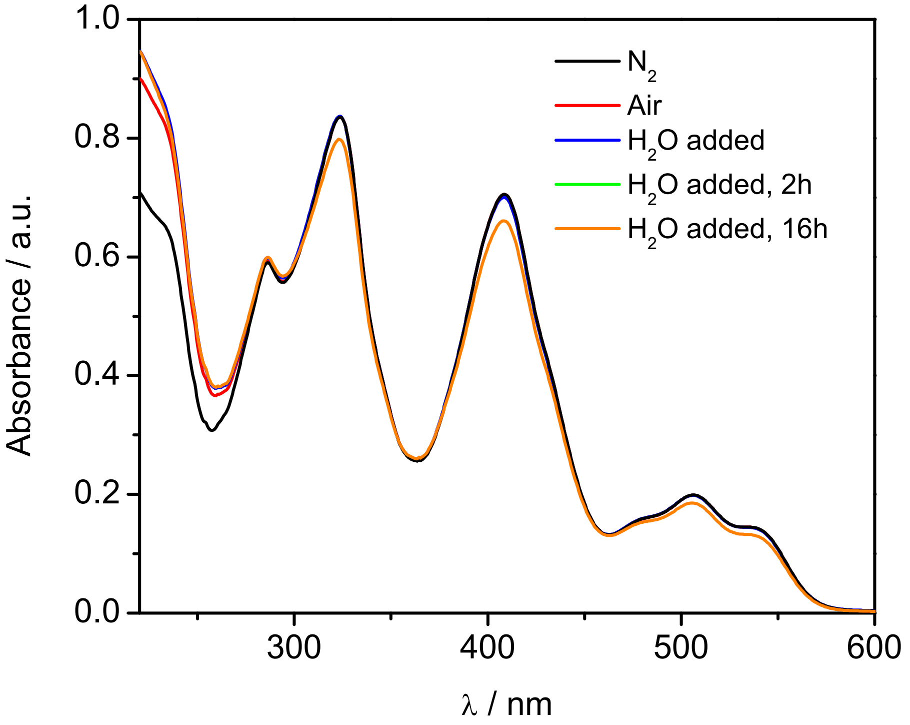

Figure S4. Comparison of the absorption spectra for a sample of Zr(bppda)$_2$ in THF under N_2, upon exposure to air, and following addition of water.

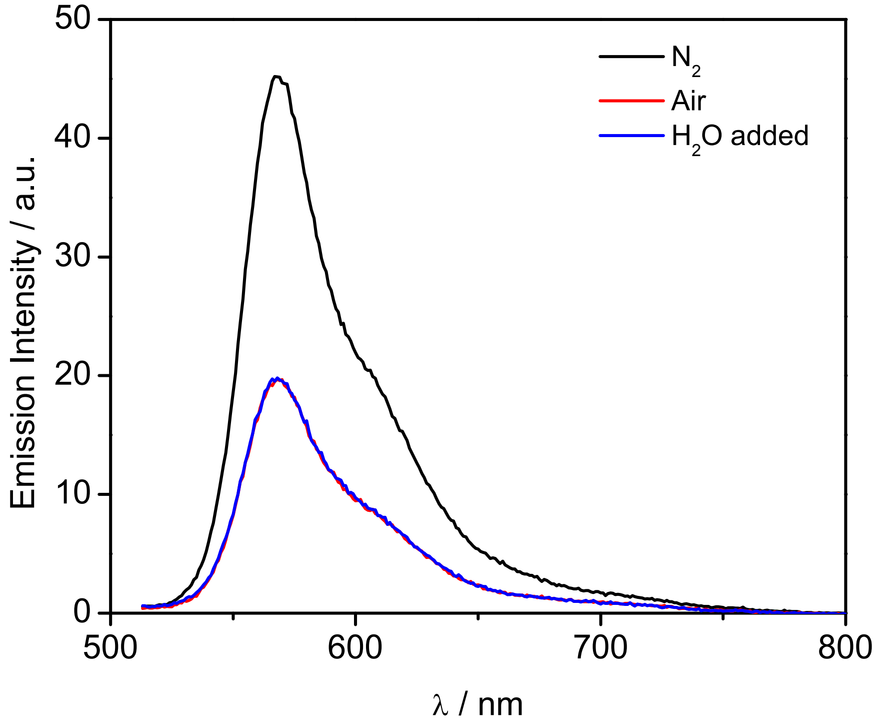

Figure S5. Comparison of the emission spectra for a sample of Zr(bppda)$_2$ in THF under N_2, upon exposure to air, and following addition of water.

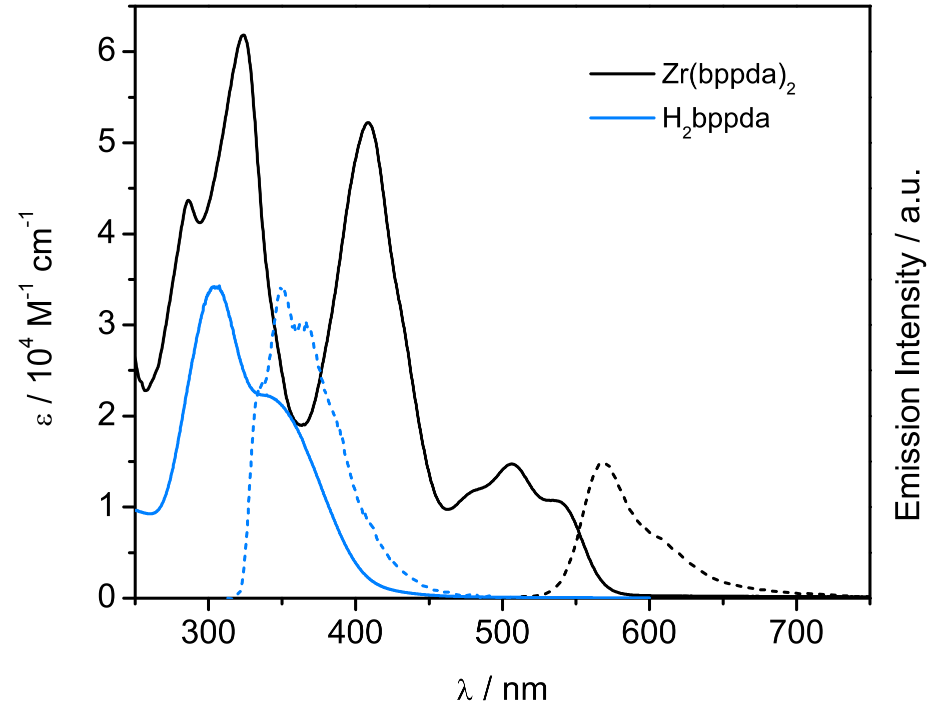

Figure S6. Comparison of the absorption and emission spectra of Zr(bppda)$_2$ and H_2bppda in THF.

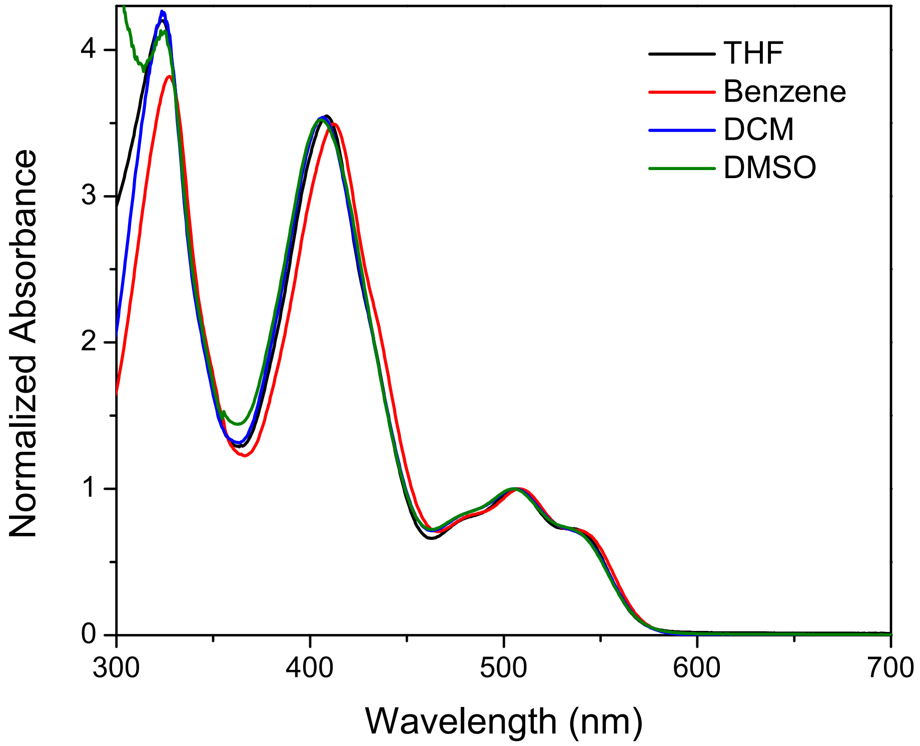

Figure S7. Comparison of the normalized electronic absorption spectra for a samples of Zr(bppda)$_2$ in different solvents.

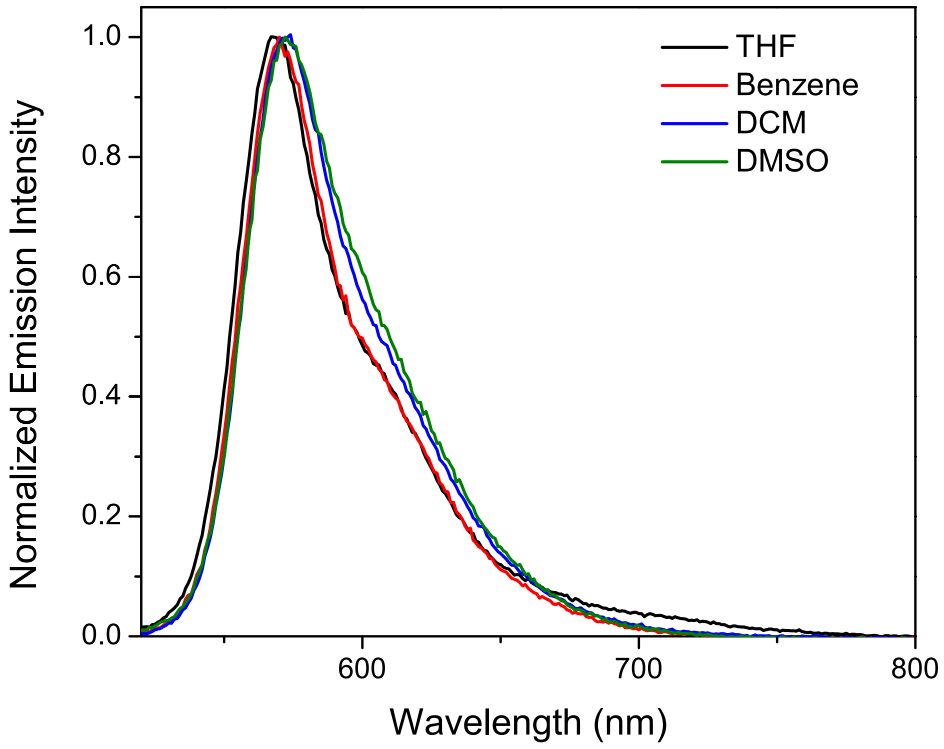

Figure S8. Comparison of the normalized emission spectra for a samples of Zr(bppda)$_2$ in different solvents under an N_2 atmosphere.

3.3 Transient Absorption Spectroscopy

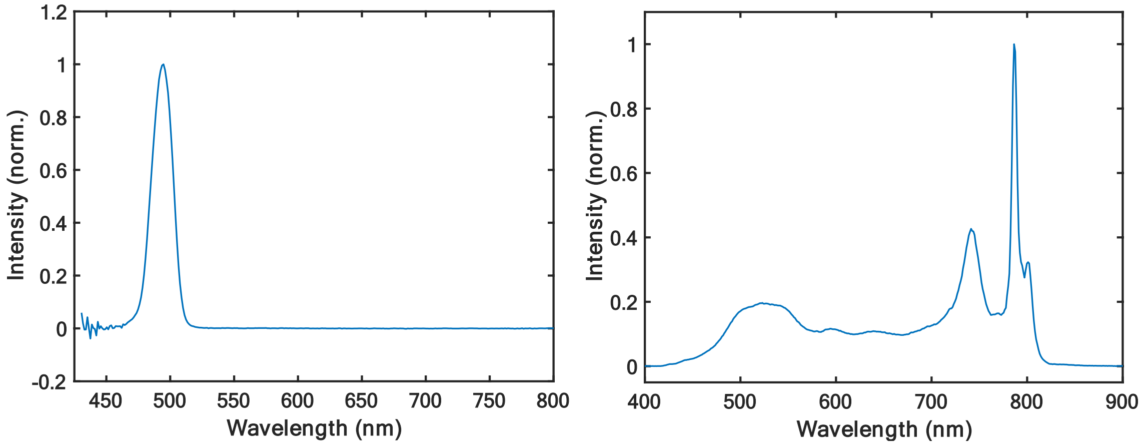

Figure S9. Left: Pump spectra for transient absorption measurements. Right: Representative spectra of white light continuum probe. White light continuum probe was generated by Ti:Sapphire crystal used for transient absorption experiment.

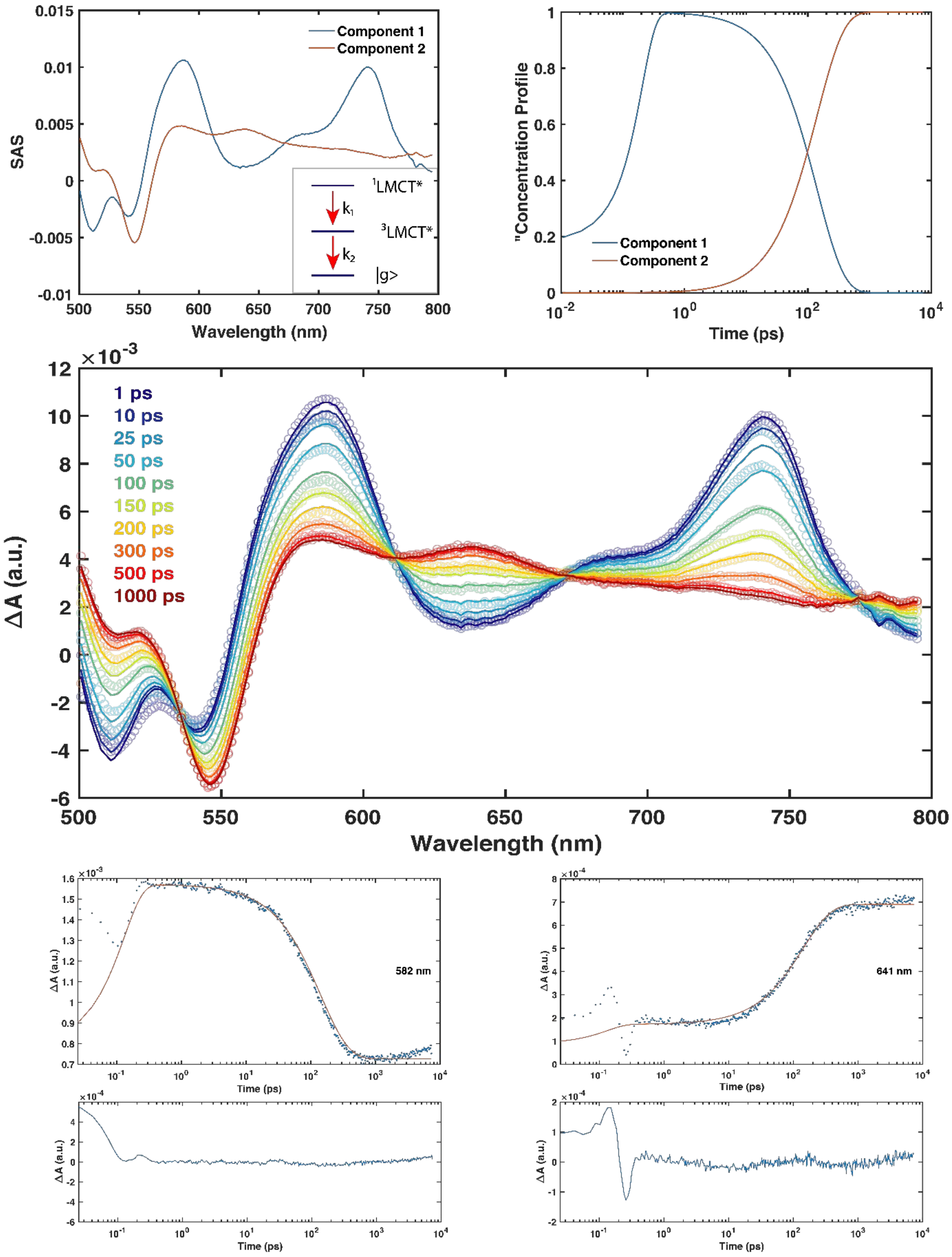

Figure S10. Top left: Species associated spectra (SAS) obtained via global analysis of the time-resolved transient absorption spectroscopic data using a model with two excited states. Top right: Relative concentration profiles of each species over time. Center: Global fit of the experimental data. Bottom: Data and fits resulting from a global analysis using a two-component kinetic scheme plotted for selected wavelengths at 582 nm and 641 nm.

4. Computational Studies

4.1 Input File Examples

Input file for geometry optimizations

```
!RKS B3LYP TightSCF Zora zora-def2-svp sarc/j Normalprint CPCM(thf) UseSym Opt

%pal nprocs 12 end
%maxcore 4000
%basis newgto Zr "sarc-zora-tzvp" end
        newgto N "zora-def2-tzvp" end
       end
%method
SymThresh 5.0e-2
end

*xyz 0 1
Coordinates from X-ray crystallography
*
```

Input file for TD-DFT calculations

```
!RKS B3LYP TightSCF ZORA zora-def2-svp sarc/j Normalprint CPCM(thf)

%pal nprocs 12 end
%maxcore 4000
%basis newgto Zr "sarc-zora-tzvp" end
        newgto N "zora-def2-tzvp" end
       end
%tddft nroots 50
        donto true
        ntostates 1,2,3,4,5,6,7,8,9,10
        tda false
        upop true
        triplets true
        irootlist 1,2,3,4,5,6,7,8,9,10
        trootlist 1,2,3,4,5,6,7,8,9,10
end

*xyz 0 1
Coordinates from geometry optimization
*
```

4.2 Additional Results from TD-DFT Calculations

Table S2. Vertical electronic excitation energies and main excitations contributing to the absorption bands of $Zr(bppda)_2$ obtained by TD-DFT calculations. Only states with a calculated oscillator strength larger than 0.05 are shown.

TD-DFT State	Energy / cm^{-1} (λ / nm)	f_{osc}	Excitations (weight)[a,b]	Character (% Zr)
1	18,696 (534.9)	0.06	155 → 156 (0.95)	^{1}IL/^{1}LMCT (25%)
2	18,696 (534.9)	0.06	154 → 156 (0.95)	^{1}IL/^{1}LMCT (25%)
4	23,156 (431.9)	0.16	155 → 157 (0.97)	^{1}IL (0%)
5	23,156 (431.9)	0.16	154 → 157 (0.97)	^{1}IL (0%)
10	25,061 (399.0)	0.44	155 → 159 (0.39)<br>154 → 158 (0.39)<br>152 → 157 (0.09)	^{1}IL (3%)
14	28,355 (352.7)	0.16	151 → 156 (0.67)<br>152 → 157 (0.30)	^{1}IL/^{1}LMCT (17%)
19	30,144 (331.7)	0.58	152 → 157 (0.56)<br>151 → 156 (0.26)<br>153 → 160 (0.07)	^{1}IL (9%)
20	30,731 (325.4)	0.46	150 → 156 (0.61)<br>155 → 160 (0.16)<br>152 → 158 (0.11)	^{1}IL/^{1}LMCT (21%)
21	30,731 (325.4)	0.46	149 → 156 (0.61)<br>154 → 160 (0.16)<br>152 → 159 (0.11)	^{1}IL/^{1}LMCT (21%)
26	33,898 (295.0)	0.42	153 → 160 (0.69)<br>147 → 156 (0.20)	^{1}IL/^{1}LMCT (31%)

[a]only excitations with a weight larger than 0.05 are shown. [b]HOMO 154, 155 (degenerate), LUMO 156.

4.3 xyz-Coordinates

Zr(bppda)$_2$ – S$_0$

Zr	-0.00000156742419	-0.00000085971839	0.00000010097232
N	0.92754515905204	0.92754732531119	2.00364237693599
N	-0.92754842873024	-0.92754697512825	2.00364368500253
N	1.58248436252523	1.58248480456078	-0.53035816608744
N	-1.58248907454197	-1.58248594627282	-0.53035589414769
N	-0.92754809983471	0.92754549639387	-2.00364298436022
N	0.92754635826771	-0.92754841709807	-2.00364262507065
N	-1.58248773914530	1.58248581823713	0.53035667898639
N	1.58248598761604	-1.58248624291939	0.53035748392652
C	0.50153450666044	0.50153437807084	3.27039141996543
C	-0.50153544432890	-0.50153470821216	3.27039215956423
C	-0.98924860358118	-0.98924955763393	4.49793995588650
C	-0.49597531517384	-0.49597966335579	5.70492941323248
C	0.49598137193322	0.49597563606308	5.70492870482835
C	0.98925101010468	0.98924755214028	4.49793855671029
C	1.84550699071481	1.84550686847365	1.83911182269861
C	2.21578423015320	2.21578308957582	0.53229494282151
C	3.15341669018322	3.15341269705376	0.05299277426297
C	3.08606688432335	3.08606345886964	-1.34700614937713
C	2.11381082402874	2.11381032272219	-1.65298322823513
C	-1.84551039953716	-1.84550664215600	1.83911433747013
C	-2.21578860009770	-2.21578346673151	0.53229789286622
C	-3.15342129630471	-3.15341332371610	0.05299663391699
C	-3.08607214826587	-3.08606482076069	-1.34700237281009
C	-2.11381609423014	-2.11381201769113	-1.65298041199160
C	-0.50153464669909	0.50153390626688	-3.27039150490025
C	0.50153571694159	-0.50153481269036	-3.27039135743884
C	0.98925269834312	-0.98924691900897	-4.49793874212411
C	0.49598314645986	-0.49597437778939	-5.70492865388623
C	-0.49597397880396	0.49598048081941	-5.70492884080983
C	-0.98924768744332	0.98924937884685	-4.49793912726853
C	-1.84550987654526	1.84550532969156	-1.83911357730686
C	-2.21578747447262	2.21578288797627	-0.53229718868078
C	-3.15341936394508	3.15341363099832	-0.05299616870911
C	-3.08606936105958	3.08606629693380	1.34700283842901
C	-2.11381372966714	2.11381313389198	1.65298112569899
C	1.84550872732906	-1.84550755760482	-1.83911254821510
C	2.21578628115791	-2.21578391796209	-0.53229583188826
C	3.15341951751143	-3.15341289737604	-0.05299396110335
C	3.08606988394738	-3.08606392498235	1.34700497016571
C	2.11381316943250	-2.11381154291817	1.65298236690090

H	-1.75785789854001	-1.75785861109903	4.51950094530856
H	-0.88450648536255	-0.88451275289436	6.64535189882856
H	0.88451561059592	0.88450696537240	6.64535067133527
H	1.75786072617965	1.75785620137644	4.51949848883918
H	2.33484821176102	2.33484604952171	2.68390424688787
H	3.79033126365257	3.79032491025765	0.66032959546698
H	3.66207522305635	3.66206979534245	-2.06517443574224
H	1.79216281394272	1.79216261093745	-2.63723143125061
H	-2.33485076644985	-2.33484556285717	2.68390739759468
H	-3.79033545859493	-3.79032533107856	0.66033405982586
H	-3.66208066748665	-3.66207165002608	-2.06517011061862
H	-1.79216815917471	-1.79216517516834	-2.63722891395478
H	1.75786282332975	-1.75785519861492	-4.51949901233749
H	0.88451781103555	-0.88450483140594	-6.64535078941316
H	-0.88450509769587	0.88451410765302	-6.64535115083185
H	-1.75785721774280	1.75785820536138	-4.51949974349312
H	-2.33485003952311	2.33484444378850	-2.68390663058462
H	-3.79033347538008	3.79032554314418	-0.66033377013317
H	-3.66207710878922	3.66207406821524	2.06517044035283
H	-1.79216551229923	1.79216690323123	2.63722975169100
H	2.33485035942330	-2.33484596031779	-2.68390517562104
H	3.79033450228306	-3.79032447097214	-0.66033100202139
H	3.66207877490929	-3.66206992012906	2.06517307548557
H	1.79216518001628	-1.79216424080915	2.63723068755489

$Zr(bppda)_2$ – T_1

Zr	-0.00000393022113	-0.00000424691505	0.00282552105034
N	0.93440820581331	0.93441052034876	2.00949250969238
N	-0.93441679407617	-0.93442046833400	2.00949317229148
N	1.57984275927818	1.57984716632826	-0.51934422340406
N	-1.57985607472988	-1.57984427366639	-0.51934234128364
N	-0.92808808357881	0.92808775453252	-1.98735548714549
N	0.92808827028672	-0.92809499376646	-1.98735433657919
N	-1.59511381627178	1.59511474867081	0.55185231248294
N	1.59512239004456	-1.59510857304940	0.55185418201816
C	0.51495065372976	0.51494921620874	3.22294864218779
C	-0.51495779417904	-0.51496238222982	3.22294709216159
C	-0.99936787010173	-0.99937591013742	4.47802503261509
C	-0.50483089829437	-0.50483993136784	5.66234595604713
C	0.50482666959203	0.50482021077853	5.66234632350697
C	0.99936207495006	0.99935932602442	4.47802510011793
C	1.88048899715205	1.88049066495536	1.84409826926356
C	2.22399210448967	2.22399373098205	0.54251595215008
C	3.17041869844596	3.17041734153647	0.03645184640568

C	3.08889389000670	3.08888652572895	-1.34854358235649
C	2.10665802019939	2.10666030299356	-1.64520239616218
C	-1.88050260239253	-1.88050164563335	1.84409925175198
C	-2.22400537766708	-2.22399649221270	0.54251557855516
C	-3.17043273560843	-3.17041855697172	0.03644595657812
C	-3.08890935031285	-3.08887940210664	-1.34854882148575
C	-2.10667333890455	-2.10665225919615	-1.64520423360686
C	-0.50194204776421	0.50193666061791	-3.25334952294421
C	0.50193861463798	-0.50194893326874	-3.25334891243436
C	0.98883627049734	-0.98885287241053	-4.48180784022455
C	0.49592050145771	-0.49593733379895	-5.68918143501507
C	-0.49592888518599	0.49591798956594	-5.68918176518311
C	-0.98884246116484	0.98883717297547	-4.48180869292560
C	-1.84777345605904	1.84778012537372	-1.82361595879040
C	-2.22172927546577	2.22173706339167	-0.52062312796507
C	-3.16462542439776	3.16464344190705	-0.05367339073303
C	-3.10671296603462	3.10672254232288	1.34471862311740
C	-2.13482198499213	2.13482925689150	1.66388528006525
C	1.84778403925067	-1.84777675570277	-1.82361345373255
C	2.22174408972151	-2.22172687709859	-0.52061993701296
C	3.16465228628074	-3.16462034518059	-0.05366873168004
C	3.10674372259887	-3.10669325498448	1.34472316653305
C	2.13484058613480	-2.13481178296858	1.66388825398552
H	-1.76731522534855	-1.76732528834877	4.50327209919028
H	-0.88464151314067	-0.88465352397797	6.60979124106952
H	0.88463913125293	0.88463169961632	6.60979174382813
H	1.76730948208702	1.76730879378778	4.50327354147298
H	2.36725377523778	2.36725355827002	2.68715123640475
H	3.81083992354777	3.81083627470048	0.63538171115888
H	3.65671061289524	3.65669697044039	-2.07999089695915
H	1.78300686884255	1.78301060104791	-2.62797930961639
H	-2.36726684797857	-2.36726545596675	2.68715107818684
H	-3.81085354923670	-3.81084050670602	0.63537291845940
H	-3.65672720977094	-3.65668551963267	-2.07999863834709
H	-1.78302372737515	-1.78299746241171	-2.62797979710641
H	1.75750683078266	-1.75752885542263	-4.50314690307466
H	0.88461936007813	-0.88464131559806	-6.62955738340403
H	-0.88462982746293	0.88461893739784	-6.62955805805151
H	-1.75751351319206	1.75751265199502	-4.50314872207601
H	-2.33583131198004	2.33584324013870	-2.66999403915745
H	-3.79694267964953	3.79696777464778	-0.67065645779854
H	-3.68715608650046	3.68716626734965	2.05601389326791
H	-1.82246289857098	1.82246653873405	2.65466007440907
H	2.33584869549386	-2.33583482465369	-2.66999034984663

H	3.79697610185318	-3.79693909605985	-0.67065079351245
H	3.68719648526349	-3.68712609444713	2.05601938588367
H	1.82247944570670	-1.82244983603507	2.65466259370592

5. References

(1) Brouwer, A. M. Standards for Photoluminescence Quantum Yield Measurements in Solution (IUPAC Technical Report). *Pure Appl. Chem.* **2011**, *83*, 2213–2228.

(2) Würth, C.; Grabolle, M.; Pauli, J.; Spieles, M.; Resch-Genger, U. Relative and Absolute Determination of Fluorescence Quantum Yields of Transparent Samples. *Nat. Protoc.* **2013**, *8*, 1535–1550.

(3) Fischer, M.; Georges, J. Fluorescence Quantum Yield of Rhodamine 6G in Ethanol as a Function of Concentration Using Thermal Lens Spectrometry. *Chem. Phys. Lett.* **1996**, *260*, 115–118.
